# Recent Advances in Fire Safety of Carbon Fiber-Reinforced Epoxy Composites for High-Pressure Hydrogen Storage Tanks

**DOI:** 10.3390/polym16233343

**Published:** 2024-11-28

**Authors:** Omar Dagdag, Hansang Kim

**Affiliations:** Department of Mechanical Engineering, Gachon University, Seongnam 13120, Republic of Korea; omardagdag@gachon.ac.kr

**Keywords:** carbon fiber, epoxy resins, nanomaterials, composite materials, fire safety, hydrogen storage tanks

## Abstract

The increasing use of hydrogen as a clean energy carrier has underscored the necessity for advanced materials that can provide safe storage under extreme conditions. Carbon fiber-reinforced epoxy (CFRP) composites are increasingly utilized in various high-performance applications, including automotive, aerospace, and particularly hydrogen storage tanks, due to their exceptional strength-to-weight ratio, durability, excellent corrosion resistance, and low thermal conductivity. However, the inherent flammability of epoxy matrices poses significant safety concerns, particularly in hydrogen storage, where safety is paramount. This review paper provides a comprehensive overview of the recent progress in enhancing the fire safety of CFRP. The focus is on innovative strategies such as developing novel flame-retardant (FR) additives, intumescent coatings, and nanomaterial reinforcements. It analyzes the effectiveness of these strategies in improving the fire performance of CFRP composites, including their flame retardancy, smoke suppression, and heat release rate reduction. The review paper also explores the application of fire modeling tools to predict the fire behavior of CFRP composite hydrogen storage tanks under various fire scenarios. Additionally, the review discusses the implications of these advancements on the performance and safety of hydrogen storage tanks, highlighting both the progress made and the challenges that remain.

## 1. Introduction

The growing need for clean and sustainable energy options has spurred the advancement of hydrogen as a key alternative energy carrier. Hydrogen storage, especially in high-pressure tanks, plays a vital role in hydrogen energy systems, facilitating its safe transportation and application in areas like fuel cell vehicles and industrial energy systems [1]. In recent years, hydrogen has garnered significant interest as a clean fuel option. Consequently, various hydrogen storage methods have been developed to meet this growing demand. Storage tanks play a crucial role in advancing hydrogen storage and transportation technologies. Currently, pressure hydrogen storage technology is the most effective solution to meet requirements, with type III and type IV hydrogen tanks being the most commonly used pressure storage options [2].

Among the different materials utilized for hydrogen storage, carbon fiber-reinforced epoxy composites (CFRPs) have become a preferred option due to their outstanding strength-to-weight ratio, excellent chemical stability, and capacity to endure the high pressures necessary for effective storage (e.g., up to 700 bar) [3,4]. Despite their advantages, the use of CFRPs in hydrogen storage tanks presents several challenges. A major issue is their fire safety performance, especially in situations where the tank is subjected to high temperatures or flames [5,6]. Failure of CFRPs in these conditions can result in severe outcomes, such as tank rupture and hydrogen leakage, which may lead to explosions or fires [7,8]. Consequently, fire safety regulations for hydrogen storage systems require stringent adherence, prompting the need for the development of advanced composite materials and manufacturing methods that improve fire resistance while preserving mechanical integrity [9,10,11].

Recent studies have concentrated on enhancing the fire retardancy of CFRPs by incorporating flame-retardant additives into their epoxy matrix, applying protective coatings, and investigating advanced manufacturing techniques to reduce defects like porosity, which can negatively impact fire resistance. Additionally, understanding the interplay between material performance, structural design, and real-world conditions, such as high-pressure storage, continues to be a vital area of research.

This review seeks to offer a thorough overview of the latest developments in improving the fire safety of CFRPs used in high-pressure hydrogen storage tanks. It covers the challenges related to fire retardancy and the influence of manufacturing techniques on performance enhancement. Additionally, it highlights emerging trends and future research directions aimed at meeting the increasing demand for safer hydrogen storage solutions.

## 2. CFRPs for High-Pressure Hydrogen Storage Tanks Applications

The use of epoxy-reinforced carbon fiber (CFRP) composites for hydrogen storage tanks poses unique fire safety challenges. These tanks, which are essential for hydrogen vehicles, must withstand extreme temperature and pressure conditions, especially in the event of a fire. Fire safety becomes a critical factor because carbon fiber composites, although lightweight and strong, can rapidly decompose under heat, releasing flammable gases and reducing mechanical strength. Studies show that the pyrolysis of carbon fiber–epoxy composites in hydrogen storage tanks requires optimization of kinetic parameters to improve fire resistance [12]. In addition, new reinforcement methods, such as the addition of flame retardants, can improve fire safety without compromising the mechanical properties of the materials [13]. These advances are crucial to ensure the safety of pressure vessels in industrial and transportation applications, where fire risk is a major concern. The fire safety of CF-reinforced epoxy composites for hydrogen tanks can be improved through a combination of kinetic modeling and reinforcement with flame retardants while maintaining high mechanical performance.

### 2.1. Safety Evaluation of CFRP High-Pressure Hydrogen Storage Tanks

The safety assessment of CFRP high-pressure hydrogen storage tanks is critical to ensuring their dependable use in hydrogen-powered vehicles and energy systems. CFRP tanks have advantages such as a high strength-to-weight ratio and corrosion resistance, making them suitable for hydrogen storage at pressures up to 700 bar. However, their safety assessment must consider critical issues such as hydrogen permeation, material degradation under cyclic loading, and impact resistance. Additionally, the potential for hydrogen embrittlement in the liner material, leak detection, and the effectiveness of pressure relief devices are all important considerations. Comprehensive safety evaluations aim to reduce the risks associated with leaks, fires, and explosions, ensuring that CFRP hydrogen tanks meet stringent safety standards while remaining operational in demanding environments. A number of studies [14] have examined hazard risk analysis, fire resistance, and thermal response modeling, including consequence evaluation of hydrogen storage tanks under varied fire conditions. In a confined fire test, Zheng et al. [15] showed that the internal pressure increase rate in type III tanks varied only by 6% when both air and hydrogen were utilized as filling media independently. In a bonfire test on 7.5 L type IV tanks, Hupp et al. [16] discovered that the fire resistance duration decreased with increasing initial filling pressure with increased fire impingement area with increasing tank surface temperature. The effects of an unintentional explosion of a type III hydrogen retention tank holding 165 L and 35 MPa in the fire were assessed by Shen et al. [17]. In an experimental investigation of the residual strength of a nitrogen tank following flame exposure, Tamura et al. [18] discovered that the tank’s burst level was more than double the original filling pressure. However, since it is extremely challenging and expensive to verify the ultimate pressure-carrying capability of hydrogen containers under different operating circumstances, very few experimental investigations have been carried out. Furthermore, it is challenging to optimize tank layout and develop pertinent standards since no quantitative investigation of the elements determining the final pressure-bearing capacity of hydrogen retention tanks has been carried out. The maximum pressure-bearing capacity of hydrogen storage containers under various fire scenarios is thoroughly experimentally analyzed by Wang et al. [14], with a focus on the impact of fire damage, including the flame exposure period, on pressure response characteristics. To simulate real-world accident conditions, this study takes a systematic approach that includes hydraulic bursts and bonfire tests. One of the study’s significant findings is the development of a grey correlation analysis model that quantitatively evaluates the influence of various factors on the tanks’ critical failure pressure under fire conditions. This novel approach not only improves our understanding of tank behavior during emergencies but also provides valuable insights for optimizing tank design and developing effective emergency response strategies, resulting in improved safety measures for hydrogen storage systems in hydrogen fuel cell vehicles. The main findings of this work reveal that the actual burst pressure of the type III hydrogen storage tank of 48 L and 70 MPa tested at room temperature was 209.8 MPa, which is approximately 300% of the nominal working pressure (NWP), indicating a substantial safety margin above the minimum design requirements. The study also discovered that under fire conditions, the tank’s critical failure pressure decreased significantly by approximately 63.1%, highlighting the vulnerability of hydrogen storage systems to thermal damage. However, after cooling, the thermally damaged tank’s residual burst pressure was only 14.5% lower than its original capacity, indicating that some structural integrity was preserved post-exposure. Furthermore, the study established a grey correlation analysis model that quantitatively assessed the influence of various factors, such as wall thickness and flame exposure time, on the tanks’ pressure-bearing performance, providing critical insights for improving safety measures and designing strategies for hydrogen storage systems in hydrogen fuel cell vehicles. Zhou et al. [11] investigated the thermal response and jet flame behavior of a hydrogen storage tank, providing useful analysis and predictions about flame length and thermal properties. The key findings about the thermal response of the hydrogen storage tank in fire scenarios show that the tank’s maximum wall temperature reached 860 °C before hydrogen release, with a critical internal pressure of 77.4 MPa, or approximately 110% of its nominal working pressure. The study found that thermal radiation from jet flames poses significant risks, with a maximum intensity of 5 kW/m^2^ measured at a distance of 2 m, exceeding the maximum tolerable exposure for humans. During the first 15 s of hydrogen release, the tank experienced a rapid pressure drop of more than 1 MPa/s, resulting in the formation of a large jet flame. Furthermore, the fire typically progressed from localized to engulfing, with the tank enduring localized fire for approximately 540 s before activating the thermal pressure relief device (TPRD).

### 2.2. Explosion Mechanism and Consequences of CFRP High-Pressure H_2_ Tanks in Fire

The explosion mechanism and consequences of CFRP high-pressure hydrogen tanks in fires raise serious safety concerns. When exposed to fire, the high-pressure hydrogen in the tanks can rapidly increase in temperature, resulting in over-pressurization. If the pressure exceeds the tank’s structural limits, it can cause a catastrophic rupture. The fire weakened the CFRP layers, hastening tank failure. This can result in a hydrogen release, which can ignite and cause a powerful explosion, typically through jet fires or vapor cloud explosions (VCE). The consequences include thermal radiation, shock waves, and the projection of high-velocity CFRP fragments, all of which pose serious risks to nearby structures and people. Understanding these mechanisms is critical for creating fireproof designs and effective safety protocols.

The rupture of a high-pressure hydrogen tank during a vehicle fire event releases a significant amount of mechanical and chemical energy, which dissipates in four primary forms: destruction of the vessel, heat transfer to the surrounding environment through combustion and thermal radiation, expansion work manifested as a blast wave, and kinetic energy from flying fragments. Genova et al. [19] highlighted these energy dissipation mechanisms, while Halm et al. [20] developed a finite element model to predict the failure time of type IV tanks, noting that the thermal behavior of polymeric liners differs from that of aluminum liners in type III tanks, with the polymer melting at 135 °C potentially leading to hydrogen leakage. Zalosh observed that a type III hydrogen tank (88 L, NWP 35 MPa) ruptured after 12.3 min of fire exposure, producing a fireball with a diameter of approximately 24 m and propelling SUV fragments up to 107 m away while measuring an overpressure of 140 kPa at 1.2 m. Similarly, Shen et al. [17] evaluated a type III tank (165 L, NWP 35 MPa) and found their thermal radiation and debris distance estimates to be consistent with observed accident data. Tschirschwitz et al. [21] conducted bonfire tests on LPG cylinders, revealing an average burst pressure of 7.93–8.87 MPa and overpressures of 5–15 kPa at 5 m, with a maximum of 27 kPa. Unlike the rapid chemical explosion of compressed hydrogen, LNG explosions are characterized by boiling liquid expanding vapor explosions (BLEVE), which do not produce a blast wave due to the slower liquid flashing process. Furthermore, Molkov and colleagues [22] explored blast wave decay correlations for hydrogen tank ruptures, contributing to the understanding of hydrogen safety engineering. Despite the application of high-pressure hydrogen storage (>35 MPa) in hydrogen fuel cell vehicles (HFCVs), there remains a lack of experimental data on the explosion characteristics of type III tanks under fire conditions, highlighting the need for further research and validation of consequence evaluations and simulation results. A study by Wang et al. [10] aimed to obtain insights into the explosive behavior of high-pressure hydrogen storage tanks in fire scenarios, providing valuable experimental data that enhances our understanding of hydrogen safety.

**Figure 1:**
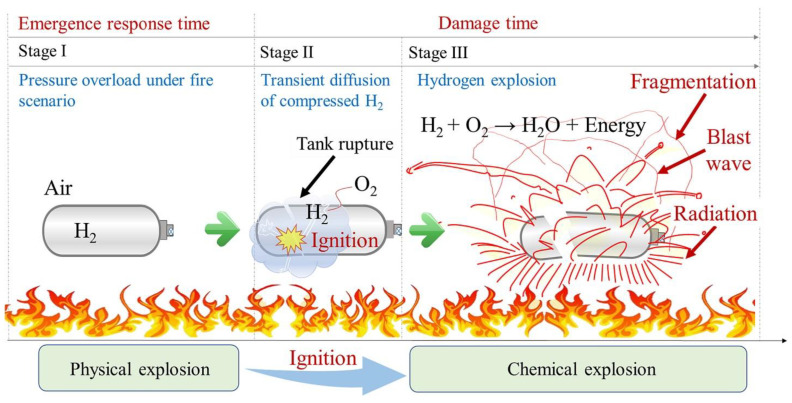
Explosion mechanism of a fuel vehicle tank rupture during a fire [10]. Copyright 2023 Elsevier.

This study looked into the explosion mechanisms and hazardous consequences of high-pressure hydrogen storage tanks, specifically type III tanks. It discovered that under fire conditions, the tank’s average bearing capacity decreases by approximately 60.3%, with a rapid internal pressure increase resulting in a deflagration lasting about 2 s and a fireball diameter of 4.48 m. It emphasizes that fragments can be propelled up to 46.0 m, with peak overpressure reaching 875.33 kPa just 2 m away from the explosion site, posing significant risks to nearby structures and people. A modified physical model of a high-pressure hydrogen retention tank explosion is shown in Figure 1. A common high-pressure hydrogen container explosion may be broken down into three phases based on the steps as well as chronological order: stage I is the physical explosion of the tank, stage II is the igniting stage, and stage III is the hydrogen chemical explosion. These stages all happen practically immediately. Due to the effect of an external heat source, the average kinetic energy, along with the thermal motion rate of the hydrogen molecules contained in the tank, rose throughout stage I, which caused a steady rise in internal pressure. At the same time, fire damage decreased the carbon fiber composites’ mechanical qualities. The high-pressure hydrogen broke free from the wall’s constraint and expanded quickly, releasing a significant amount of physical energy right away, until the coupling effect of the external high temperature alongside the continuously rising internal pressure on the material damage led to the formation of a burst [7]. The tank burst as a physical explosion since the hydrogen in the bottle, along with the ambient air, was not coming into contact throughout this procedure. The separation of hydrogen and oxygen within and outside the cylinder prior to its rupture is another factor that makes stage I a physical explosion. Therefore, rather than being a chemical reaction, this event might be seen as a physical tank rupture brought on by high internal pressure. When the tank burst, the high-pressure hydrogen rapidly grew into the atmosphere throughout stage II, often known as the ignition stage. Upon the contact surface, hydrogen was ready to use with the air due to the high-speed blast wave that was traveling through the air from the tank, which caused ignite when it came into contact with an open flame source [22]. One may consider the stage III hydrogen–air deflagration to be a chemical explosion. A huge fireball, mushroom cloud, as well as other phenomena, were quickly formed as a result of the release of a significant quantity of chemical energy. The chaotic non-premixed combustion had almost little impact on the blast wave strength until it faded and moved away from the tank [23]. In addition to releasing a significant amount of energy, the explosion simultaneously created a fireball, blast wave, as well as debris, severely damaging the nearby persons and equipment [22].

### 2.3. Relationship Between Fire Resistance Duration and Filling Pressure in Hydrogen Tanks

The relationship between fire resistance duration and filling pressure in hydrogen tanks is crucial for evaluating safety during fire incidents. In a bonfire test involving 7.5 L type IV tanks, those filled with hydrogen at 700 bar exhibited a fire resistance time of approximately 5.5 min before bursting, while tanks filled at 350 bar demonstrated a resistance time of about 12.1 min, indicating a 42% increase in fire resistance with reduced pressure. Furthermore, tanks filled to 200 bar showed even longer resistance times, with leakage occurring after 17.8 min, which is only 1.17 min less than the 400 bar tank that burst. At 175 bar, leakage was observed after 8.8 min, with the internal temperature reaching 211 °C, significantly exceeding the liner’s melting temperature of 135.2 °C. These findings illustrate that as the initial hydrogen pressure decreases, the fire resistance duration increases, underscoring the critical role of filling pressure in maintaining the structural integrity of hydrogen tanks under fire exposure [16]. In tests on type III hydrogen tanks, the fire resistance duration (FRR) varied significantly with filling pressure; for example, tanks filled to an initial pressure of 32.2 MPa exhibited an FRR of 784 s before rupture, while those at 33.8 MPa had an FRR of 666 s, and tanks at 33.9 MPa showed an FRR of 596 s. This trend suggests that while higher pressures may initially enhance structural integrity, they can also lead to quicker failure once critical thresholds are reached. The average failure pressures recorded ranged from 41.1 to 41.8 MPa, indicating that although higher filling pressures can improve fire resistance, they also increase the risk of rapid failure under extreme thermal conditions [24]. Additionally, a study on a 6.8 L hydrogen storage tank filled to 30 MPa revealed a failure pressure approximately 60.3% lower under fire conditions compared to room temperature, with internal pressure rising rapidly by 52.8% within 292 s, ultimately leading to failure at 46.76 MPa. This emphasizes the need for careful consideration of filling pressures in safety assessments of hydrogen storage systems [10].

## 3. Characterization Techniques for Fire-Retardant CFRP for Hydrogen Storage Tanks

### 3.1. Combustibility Assessment by Standard Methods (Cone Calorimeter Test, UL94, LOI)

#### 3.1.1. UL 94 Test

Melting and dripping are major contributors to the spread of fire during epoxy combustion. A dripping polymer can spread fire more quickly. A horizontal as well as vertical burning test for polymer flammability is necessary to detect and control the usage of fire retardant polymers. An industrial standard for determining the flammability and ignition characteristics of polymer materials is the UL 94 test. Among the 12 flame categories of UL 94, EP materials are mainly assessed according to their V-0, V-1, along with V-2 ratings in both vertical and horizontal locations. In a 50 W blue flame that is 20 mm high, introduce the substance of interest in a homogeneous fixed dimension. Before being removed, the bottom of the material is exposed to a flame for ten seconds. The time for self-extinction is noted if the substance catches fire. The flame is extinguished and then reintroduced five times for ten seconds each time [25]. In order to receive a V-0 grade, non-inflamed particles may drop, and materials must cease burning on a vertical specimen within 10 s. When non-inflamed particles drop, and burning ceases within 30 s, the V-1 grade is achieved. The V-2 grade permits burning to cease in 30 s and dripping of flame particles [26]. This test’s dependence on the specimen’s thickness and failure to analyze the material’s inherent qualities are two drawbacks [27]. The heat gradient in the sample, along with the surface heat of the dripping polymer, is measured using an optimized UL 94 equipment with embedded thermocouples along with infrared cameras with filters for improved data collecting [28]. The findings of the UL 94 burn test’s recent numerical modeling are encouraging. This technique forecasted complicated polymer HRR along with flame spread [29].

#### 3.1.2. LOI

LOI is a test method that determines the minimum oxygen (O) concentration required to keep a material burning. The oxygen concentration is controlled in the oxygen-nitrogen (N) flow mixture. The LOI value is defined as the minimum O concentration in the O/N mixture required to maintain material combustion for 3 min or consume a 5 cm length of sample. The ASTM D2863 standard specifies a set of tests for determining LOI values. The sample is positioned vertically during the LOI test. An ideal FR material should have a LOI value greater than 25%. In general, a higher LOI indicates better flame retardancy. A material with an LOI value less than 21% is classified as “combustible”; otherwise, it is classified as “self-extinguishing”. The LOI’s calculation formula is [30]:

(1) $$\mathrm{LOI}\left(\%\right)=\frac{\left[O_{2}\right]}{\left[O_{2}\right]+\left[N_{2}\right]}\times 100$$

The flow rates of O_2_ (L/min) and N_2_ (L/min) are represented by [O_2_] and [N_2_], respectively.

#### 3.1.3. Cone Calorimetry

Both small- as well as large-scale fire testing is conducted using cone calorimeters. Cone calorimetry measures ignition, as opposed to LOI and UL-94, which assess how readily a flame may be extinguished. The sample is continuously exposed to a flame during the cone calorimetry test, which sets it apart from the other two. TTI, PHRR, THR, mass loss, char residue, smoke quantities, fire performance index, fire growth index, and gas analysis, among other metrics pertaining to the material’s burning characteristics, are all measured by the cone calorimeter [31]. ISO 5660 is the ISO standard used to determine the heat release rate in cone calorimetry [32]. For small-scale samples of polymers as well as various materials, such as wood, microscale combustion calorimetry, also called pyrolysis combustion flow calorimetry, is employed in conjunction with cone calorimetry to obtain similar flammability values [32].

Using measurements of gas flow rate and O content, HRR (heat release per unit of duration and surface area) is computed and represented in kW/m^2^. Fire properties are assessed using the HRR evolution over time, namely the peak/maximum (PHRR or HRR_max_). The HRR vs. time curve integration yields THR in kJ/m^2^. TSR, carbon monoxide and carbon dioxide concentrations, mass loss during burning, ignition interval, and combustion or extinction duration may also be determined using this test [33].

### 3.2. Thermal Degradation Analysis (TG, DTG, and DSC)

#### 3.2.1. TG Analysis

Together with FTIR, thermogravimetric analysis (TG) is utilized to identify the gas products generated during the heat degradation process. An FTIR spectrometer coupled to a TG makes up the TG-FTIR device. A characterization technique called thermogravimetric analysis weighs a specimen as it is subjected to a temperature change. The thermal behavior of the material is explained by TG information. The gases created during thermal deterioration are taken to an infrared detector for analysis in TG-FTIR. The tiny molecular gaseous breakdown products of epoxy resins are precisely identified by the FTIR signals [31].

#### 3.2.2. DTG Analysis

By measuring the test material’s rate of thermal breakdown, the DTG method makes it possible to investigate both the mechanism of the test material’s thermal breakdown and the system of action of different FRs added to EP thermosets to lessen combustibility.

#### 3.2.3. DSC Analysis

The DSC technique makes it possible to quantify the extent of thermal effects brought on by processes that take place during the thermal pyrolysis of the EP thermosets being studied.

#### 3.2.4. Py-GC-MS

Three parts make up the Py-GC-MS instrument: a pyrolizer, a GC, as well as an MS detector. Large molecules are broken up into smaller pieces when the sample is heated to 600–1000 °C. These are separated by the GC and subsequently picked up by the MS. This device detects volatile pyrolysis products that might harm human health or the environment [31].

### 3.3. SEM-EDX/EDS, NMR, Raman Spectroscopy, FTIR Spectroscopy

#### 3.3.1. SEM-EDX/EDS

SEM is frequently used to analyze the morphology of fracture surfaces and the structure of char layers in order to describe fire-retardant EPs. To ascertain the agglomeration, aggregation, and dispersion, along with phase separation of nanofillers, the fracture surface micrographs of virgin epoxy, along with their blends or composites, are examined. SEM and EDX/EDS will be used to identify the FR components in the additive. The density of distribution for every element in the EP matrix is shown in the element mapping diagram [31].

#### 3.3.2. NMR Method

By using information on the closest environments of the ^1^H, ^13^C, and ^31^P atoms in complex organic compounds, the NMR approach makes it possible to determine their chemical and conformational structure. This technique involves chemically cross-linking EP structures with other molecules or groups that contain ingredients that lessen the flammability of EP thermosets in order to illustrate the development of chemical bonds.

#### 3.3.3. Raman Spectroscopy

Identifying molecules and describing intramolecular linkages in polymers, in addition to the carbonaceous residue produced during polymer pyrolysis, are all accomplished using Raman spectroscopy. By measuring the degree of graphitization of the carbonaceous residue after pyrolysis, this approach makes it possible to assess how flame retardants affect the change in char characteristics.

#### 3.3.4. FTIR Spectroscopy

By measuring the quantities of chemicals in char along with gaseous pyrolysis products, FTIR spectroscopy is frequently used to detect compounds. Samples are compressed into tablets using KBr powder to examine the composition of char, and the transmission spectrum is examined in the infrared range (4000–500 cm^−1^). The distinctive absorption bands at 935 and 1120, along with 1230 cm^−1^ of phosphorus-containing flame retardants, may be used to identify the presence of groups like P=O, P-Ph, and P-O-Ph.

### 3.4. Emerging Techniques for Fire-Retardant CFRP in Hydrogen Storage Tanks

Advanced fire performance characterization techniques are required to assess CFRP thermal stability, flame retardancy, and overall behavior under fire conditions. These techniques aid in understanding the degradation, flame spread, smoke release, and toxicity issues in CFRPs, which are critical for improving fire safety performance in applications such as automotive, aerospace, and hydrogen storage tanks. UL 94, LOI, and cone calorimetry are the three most common methods for testing CFRP fire retardancy or flammability. There is no exact correlation between these tests. Raman spectroscopy, XPS, FTIR, as well as SEM, are also used to examine the shape and chemical makeup of char. The directors of the International Forum of Fire Research discussed the limitations of the small-scale fire tests that are now being conducted for materials in general in 2006 [34] and came to the conclusion that they may yet be improved. Many elements of fire safety risks and dangers have improved as a result of the same forum’s periodic contributions and rules [35]. For determining flammability, thermal degradation analysis techniques (TG, DTG, and DSC) are also crucial. They can be used in conjunction with other scientific methods, including chromatography, MS, as well as FTIR spectroscopy.

Using TG along with FTIR research, Tranchard et al. [36] examined the thermal breakdown of CFRP pyrolysis. The resultant gas products were analyzed, and a kinetic breakdown model was developed. There were found to be two clear pathways of heat deterioration. Hidalgo et al. [37] used FTIR analysis and a cone calorimeter test to examine epoxy resin-carbon composite fibers for hydrogen storage tanks. Cone calorimeter experiments were employed by Dao et al. to examine a number of thermal characteristics of the decomposing tank material. It was shown that when the percentage of carbon fiber in composites increases, the material’s heat resistance decreases. In both air with inert atmospheres, Régnier with Fontaine [38] used dynamic TG analysis to study carbon fiber-reinforced epoxy. The activation energy was one of the kinetic parameters that were determined. Xu et al. [39] used TGA, cone calorimeter, LOI, vertical/horizontal burning, as well as SEM examination to study the sandwich composite of carbon/epoxy laminate and foam core. Investigations were conducted into thermal behavior and a number of typical characteristic parameters. A hydrogen storage tank using CFRP that has experienced a localized fire was tested by Zheng and Ou et al. [15]. Zhang et al. [40] used TG-FTIR to study CFRP from hydrogen tanks. There was extensive discussion of a CFRP’s activation energy reaction model, including evolved gases. Several important findings emerge from the study of CFRP thermal degradation. Pyrolysis occurs predominantly between 550 K and 750 K, accounting for approximately 90% of total mass loss, indicating that significant degradation begins around 500 K. The average final residue after pyrolysis is approximately 72.42%, indicating that a significant portion of the material remains intact, potentially contributing to the formation of a thermally stable carbonaceous char that protects the underlying fibers from further decomposition. The study also found a significant variation in activation energies, ranging from 206.27 kJ/mol to 412.98 kJ/mol, with an average value of approximately 276.6 kJ/mol, indicating that the thermal degradation process is complex and likely involves multiple reaction mechanisms. The fourth reaction order model was found to be best suited for describing the pyrolysis process’s kinetics. The analysis of evolved gases revealed the presence of water (H_2_O), carbon dioxide (CO_2_), carbonyl compounds (such as acid anhydrides, ketones, or aldehydes), ε-caprolactam, alcohols, and phenol. This highlights the complexity of the degradation process and its potential implications for safety and performance in applications such as hydrogen storage tanks.

Zhang et al. [41] studied the thermal decomposition characteristics of the outer material of composite hydrogen storage tanks. Using a cone calorimeter, the authors explored how these materials behave under various heat fluxes, measuring key parameters like ignition time and heat release rate. The findings reveal important correlations and behaviors that enhance understanding of hydrogen safety and the performance of composite materials. This study concluded that the thermal decomposition behaviors of the outer composite material of hydrogen storage tanks, specifically the CFRP, were thoroughly investigated using a cone calorimeter at heat fluxes ranging from 15 to 70 kW/m^2^. The results showed that the ignition time decreased with increasing heat flux, while the heat release rate (HRR) and mass loss rate (MLR) showed distinct patterns: at heat fluxes less than or equal to 30 kW/m^2^, a single peak was observed in the MLR and HRR curves, whereas at higher fluxes, one main peak accompanied by multiple smaller peaks emerged, indicating more complex thermal behavior. The effective heat of combustion (EHC) was also measured, providing information about the energy released during decomposition. The study found that the composite material followed the thermally thick model, indicating significant internal heat transfer effects. Furthermore, correlations were found between ignition temperature, thermal response parameters, heat of gasification, and combustion heat, all of which are important in predicting the material’s performance in fire scenarios. Several experimental and numerical studies have been conducted on hydrogen storage tanks under fire conditions.

#### 3.4.1. Hydraulic Burst Pressure Test

**Figure 2:**
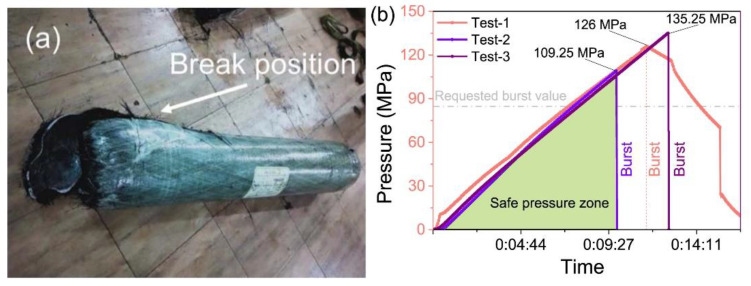
Results from the hydraulic burst test conducted on the type III tank (210 L, 35 MPa), including (**a**) an image of the break location and (**b**) the evolution of pressure over time [24]. Copyright 2022 Elsevier.

The hydraulic burst test is a critical method used to evaluate the structural integrity and safety of pressure vessels such as hydrogen storage tanks. In this procedure, the tank is filled with a liquid, typically water, and subjected to increasing internal pressure until failure occurs. The test measures the maximum pressure the tank can withstand before bursting, providing essential data on its mechanical properties and failure modes. During the test, pressure is monitored continuously, and the point of rupture is recorded, allowing for the calculation of burst pressure. This method is particularly important for type III tanks, as it helps assess their performance under extreme conditions, ensuring they meet safety standards and can safely contain high-pressure gases without catastrophic failure. The results from hydraulic burst tests are crucial for understanding the tank’s design limits and improving safety measures in hydrogen storage applications [24]. Figure 2 presents the results from the hydraulic burst test conducted on the type III tank.

#### 3.4.2. Bonfire Test of Hydrogen Storage Tank

**Figure 3:**
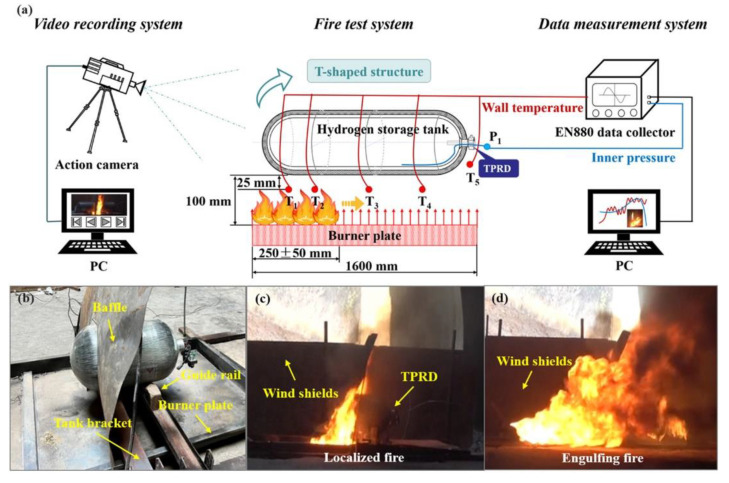
A diagram of the bonfire test system: (**a**) schematic of the hydrogen storage tank test system under fire conditions; (**b**) testing facility for conducting bonfire tests using different fire methods; (**c**) localized fire, and (**d**) engulfing fire [14]. Copyright 2024 Elsevier.

To evaluate the safety performance of hydrogen storage tanks in fire conditions, a bonfire test was conducted to assess the degradation of the tanks’ ultimate pressure-bearing capacity, as detailed by Wang et al. [14]. The bonfire test system, illustrated in Figure 3, was set up in an open shelter with a wind-proof plate to minimize wind effects. Aviation kerosene (YH–10) served as the fuel to simulate a pool fire environment typical of vehicle fires. The hydrogen storage tank was positioned horizontally 100 mm above the burner and filled to approximately 100% of its nominal working pressure (NWP). The burner was designed to create a flame that fully enveloped the tank, with its length and intensity adjustable through baffles, guide rails, and tank brackets, allowing for both localized and engulfing fire scenarios (Figure 3b). A pressure sensor monitored the tank’s internal pressure, while five Type-K thermocouples measured the outer surface temperature along the tank’s length (Figure 3a). The EN880 data collection system recorded real-time temperature and pressure data, and an action camera documented the test process. The test adhered to global technical regulations and involved igniting the fire source at different locations depending on the tank’s structure. As the test progressed, the fire source extended along the tank, reaching temperatures of 600–900 °C before transitioning to an engulfing fire, ultimately leading to pressure-bearing failure. Monitoring the thermocouples was essential for ensuring compliance and reproducibility of the test. The process of transitioning from localized to engulfing fire during the bonfire test is depicted in Figure 3c,d.

#### 3.4.3. Thermal Analysis of the Composite Materials

**Figure 4:**
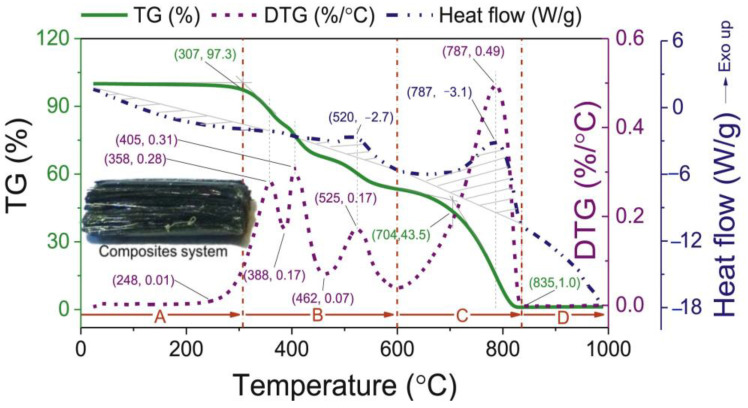
Thermal decomposition process of the EP/CF composite system [24]. Copyright 2022 Elsevier.

TGA is a critical technique employed to evaluate the thermal stability and decomposition behaviors of fire-retardant CFRP used in hydrogen storage tanks [24]. In the context of hydrogen storage, TGA involves heating composite samples, such as those made from CFs and EP, from ambient temperatures up to 1000 °C at a controlled rate, typically around 10 °C/min, in an air atmosphere. This analysis provides valuable data on the weight loss of the material as it is subjected to increasing temperatures, allowing researchers to identify key thermal degradation points and the onset of combustion. By understanding the thermal properties and degradation mechanisms of CFRP, researchers can develop enhanced fire-retardant formulations that improve the safety and performance of hydrogen storage tanks under fire conditions. The insights gained from TGA can inform the design of composite materials that not only maintain structural integrity but also exhibit improved resistance to thermal damage, thereby mitigating the risks associated with high-pressure hydrogen storage in fire scenarios. Figure 4 illustrates the decomposition process of the EP/CF composites in an air environment.

#### 3.4.4. Fire Simulation Techniques

**Figure 5:**
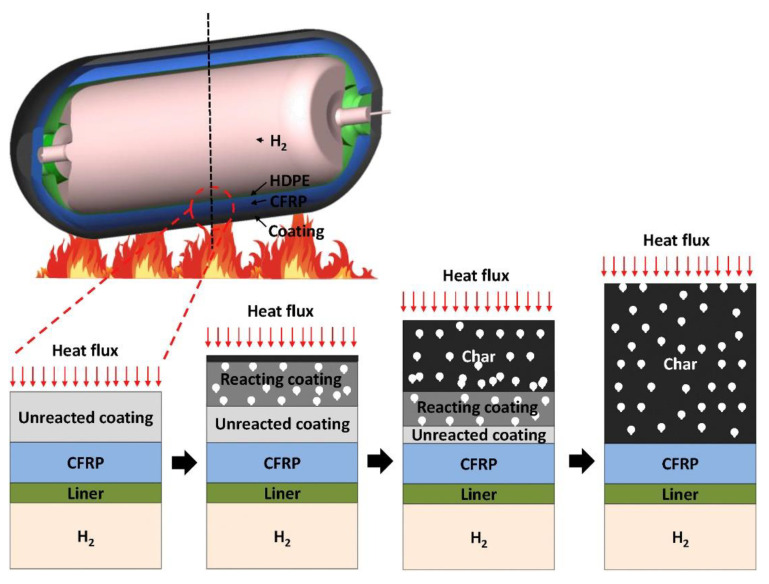
Schematic description of the problem’s simplification and physical process of intumescence [42]. Copyright 2017 Elsevier.

The fire numerical simulation in the study employs a 1-D model to analyze the thermal performance of CFRP hydrogen storage tanks coated with intumescent paint (Figure 5). This simulation is based on a three-stage approach: first, a 3D bonfire test of a bare tank is conducted to obtain the surface temperature and heat flux distribution over time, which serves as boundary conditions for the subsequent simulations. The model incorporates dynamic mesh capabilities to accurately represent the swelling behavior of the intumescent paint as it reacts to heat exposure. The total heat transfer coefficient is derived from the 3D simulation results, allowing for a detailed assessment of heat transfer mechanisms, including convective and radiative heat transfer. The validation of the 1-D model against experimental data demonstrates its reliability in predicting the fire resistance ratings of the coated tanks, highlighting the significant improvements in thermal resistance achieved through the application of intumescent coatings [42].

**Figure 6:**
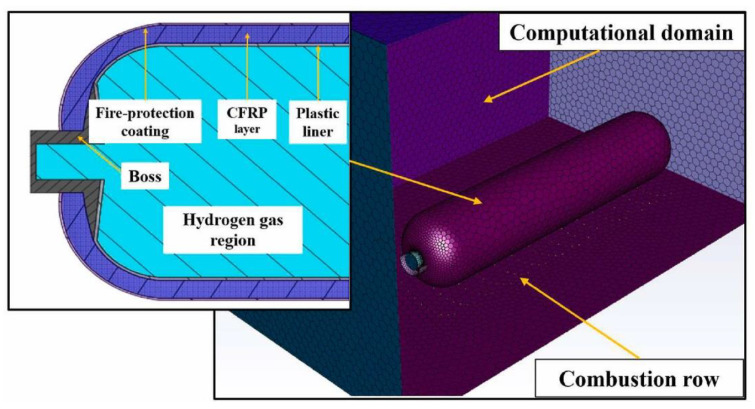
Geometric structure and computational mesh [43]. Copyright 2024 Elsevier.

The fire simulation techniques employed in another study involved the establishment of a 3D CFD (Computational Fluid Dynamics) numerical model using the AutoCAD software Fluent, which included both steady-state and transient simulations to analyze the thermal response of the hydrogen storage cylinder under fire conditions, as detailed by Li et al. [43] (Figure 6). The simulation began with a steady-state model of fire combustion, followed by a transient model to capture the heat transfer characteristics of the cylinder and its internal medium. The geometric structure of the cylinder, which included components like the CFRP layer and plastic liner, was meticulously defined, and an unstructured computational mesh was created to ensure accurate results. The mesh was optimized through grid independence checks, confirming that a configuration with 48,084 cells provided sufficient accuracy for the calculations of pressure and temperature distributions during the fire exposure.

## 4. Fire Safety of CFRPs

CFRP hydrogen storage tanks are becoming increasingly popular in the energy and transportation industries due to their lightweight design, high strength, and corrosion resistance. However, the flammability of CFRPs, particularly the polymer matrix, raises serious fire safety concerns, especially in high-pressure hydrogen storage applications where fire can result in catastrophic failure. Recent advancements in fire-safe CFRPs have focused on increasing their flame retardancy, thermal stability, and resistance to heat-induced degradation. This includes the development of advanced flame retardant additives, surface coatings, and hybrid composites that improve fire performance while preserving mechanical integrity.

### 4.1. Flame-Retardant CFRPs and Their Mechanism

#### Thermal Decomposition Mechanisms

**Figure 7:**
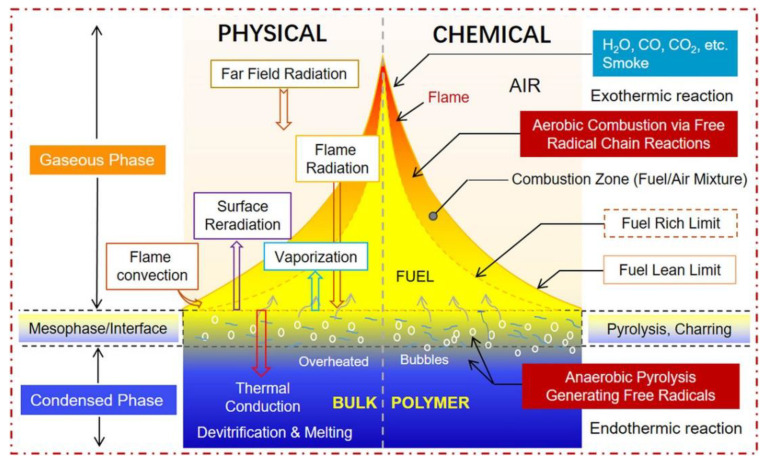
Chemical and physical processes in flame combustion of polymeric materials [51]. Copyright 2022 Wiley.

Thermal degradation of EP: When examining CFRP flammability, it is essential to comprehend the thermal decomposition process. The fiber type, along with EP formulation, has an impact on CFRP’s thermal breakdown processes. On the other hand, CFRP usually undergoes phased thermal breakdown (Figure 7).

The EP in the composite may experience thermal deterioration as the temperature rises, usually between 200 and 400 °C, which would cause the epoxy polymer chains to break down. In addition to solid char production, this may result in the emission of volatile organic chemicals and tiny molecular fragments such as phenols or low-molecular-weight hydrocarbons. Usually exothermic, this step aids in the production of heat during composite breakdown [44]. Water vapor, CO_2_, and additional hydrocarbons are among the volatile gases that are emitted as vapors or gases during the pyrolysis process. The heat and flame produced during the combustion process might be increased if these gasses continue to burn or combust [45].

Fiber decomposition: At higher temperatures, the fiber type utilized in the composite may break down. The thermal deterioration, oxidation, or combustion of CFs can occur at temperatures of 400 °C or higher.

Combustion: The solid char and volatilized gasses created during pyrolysis can catch fire and burn if the temperature is high enough. The fiber reinforcements and EP matrix burn as a result of this. Depending on the circumstances and the presence of other flammable elements, flames may emerge during the combustion process, which also produces heat, light, and smoke [46].

Char formation: The leftover carbonaceous residues from the fibers, as well as EP may begin to produce a char layer when the temperature climbs above 400–500 °C. The underlying material may be shielded from further pyrolysis and oxidation by this layer of char. One significant process that adds to CFRP’s flame resistance is the creation of the char layer [47].

As a result, flame retardants need to behave differently throughout the above-described CFRP combustion process. First, heat transmission to the composite material can be decreased by flame retardants’ ability to function as thermal insulators. This can slow down the combustion process by lowering the composite’s temperature during combustion, which will stop the EP and fibers from pyrolyzing and volatilizing [48]. Second, during combustion, flame retardants can aid in the formation of a stable char layer. By serving as a physical barrier, the char layer shields the underlying material from further heat exposure and stops volatile gasses from escaping. This can lessen the combustibility of the composite and delay the rate at which flames spread [49]. Third, by eliminating free radicals or sabotaging chemical chain processes, some flame retardants can interfere with combustion reactions in the gas phase. This may lessen the amount of fuel available for burning, which would limit the rate of combustion as a whole [50].

### 4.2. Methods for Enhancing the Flame Retardancy of CFRPs

**Figure 8:**
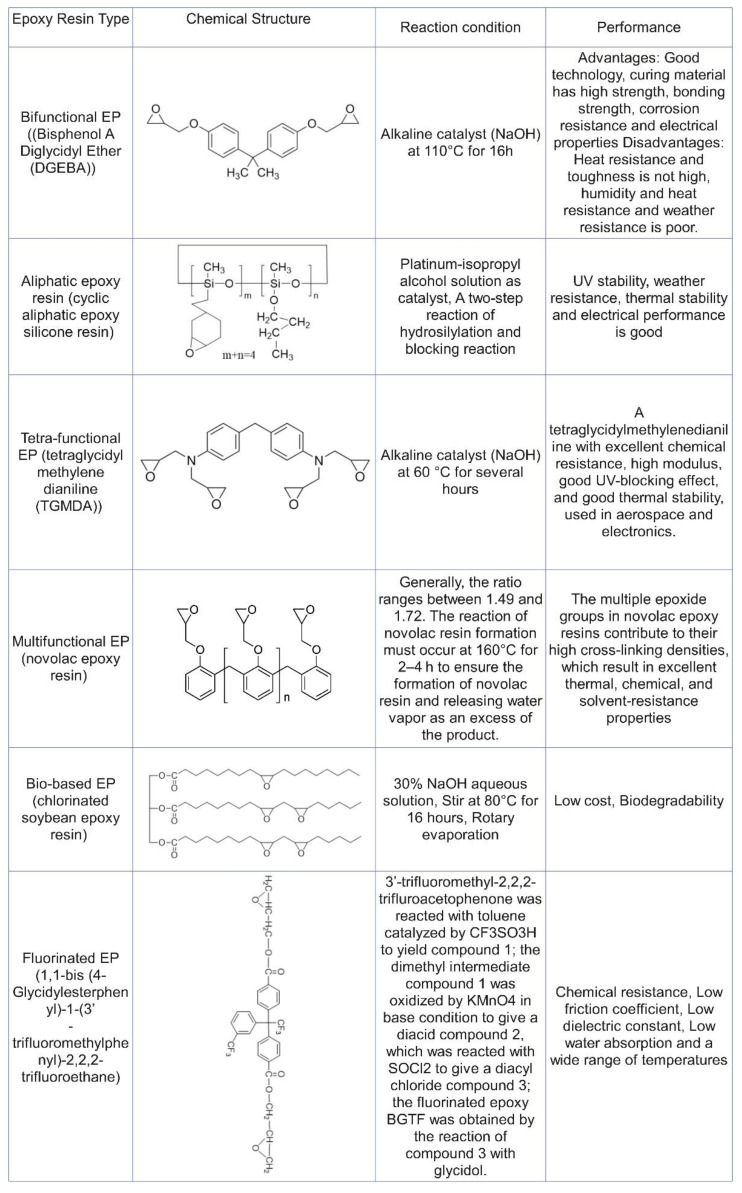
Summary of EPs [52]. Copyright 2024 Elsevier.

Epoxy resins (EPs) are low molecular weight prepolymers characterized by multiple epoxy (EP) groups, and their properties can vary significantly depending on the specific type of EP and the curing agents used. Typically, EP monomers are produced through the condensation reaction of epichlorohydrin with various compounds, including aromatic amines, diphenylmethane, polyhydroxyphenol, polyhydric alcohols, and olefin or polyolefin compounds. Additionally, another method for producing EPs involves the epoxidation of olefins using peroxy acid [52]. EP is known for its exceptional properties, including strong adhesion, resistance to wear and chemical corrosion, excellent mechanical performance, effective electrical insulation, low shrinkage, ease of processing and forming, cost-effectiveness, and the absence of by-products during curing [53]. As a result, it is extensively utilized in applications such as adhesives, electronics, electrical, aircraft, and hydrogen storage tanks. There are various types of EPs, which can be broadly categorized into aliphatic EPs, biobased EPs, fluorine-containing EPs, bisphenol-A EPs, trifunctional EPs, tetra-functional EPs, Novolac EPs, and multifunctional EPs. Figure 8 depicts the details.

**Figure 9:**
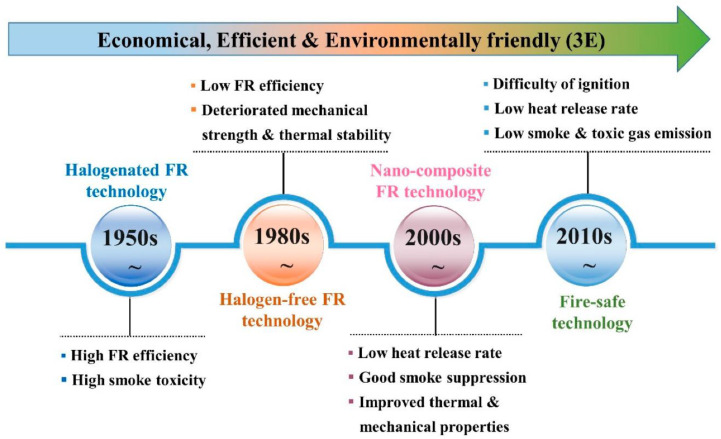
The development history of flame-retardant EPs [54]. Copyright 2024 MDPI.

Four steps are involved in the development of flame-retardant EPs (Figure 9) [54]: (1) Tetrabromobisphenol A and other halogen-containing FRs were utilized in early FR epoxy resins. Tetrabromobisphenol A produces corrosive and poisonous gases, such as hydrogen halide, that are bad for the environment and human health, even if they have a high flame retardant efficiency. Thus, in recent years, halogen-free flame-retardant EPs have gained popularity. (2) Because of their enhanced flame retardancy and environmental friendliness, halogen-free (mostly Si-, N-, P-, as well as boron-containing) flame-retardant EPs have become popular; nevertheless, their durability and/or thermal stability are often impaired. (3) Although the mechanical and thermal characteristics of flame-retardant epoxy nanocomposites are good, their flame retardancy does not satisfy the standards of UL-94 V-0 classification. (4) In addition to meeting the UL-94 V-0 standard, the latest generation of flame-retardant EPs, also referred to as fire-safe EPs, have low rates of heat release and harmful smoke emission. Low heat release rate, low smoke toxicity, and difficulty of igniting are characteristics of fire-safe epoxy resins. Low-toxicity smoke can lessen the number of people killed in fire incidents; a low heat release rate can slow the development of the flame and give individuals more time to flee; and difficulty of ignition can lessen the chance that materials will ignite. Therefore, the next development in flame-retardant EPs is fire-safe EPs.

**Figure 10:**
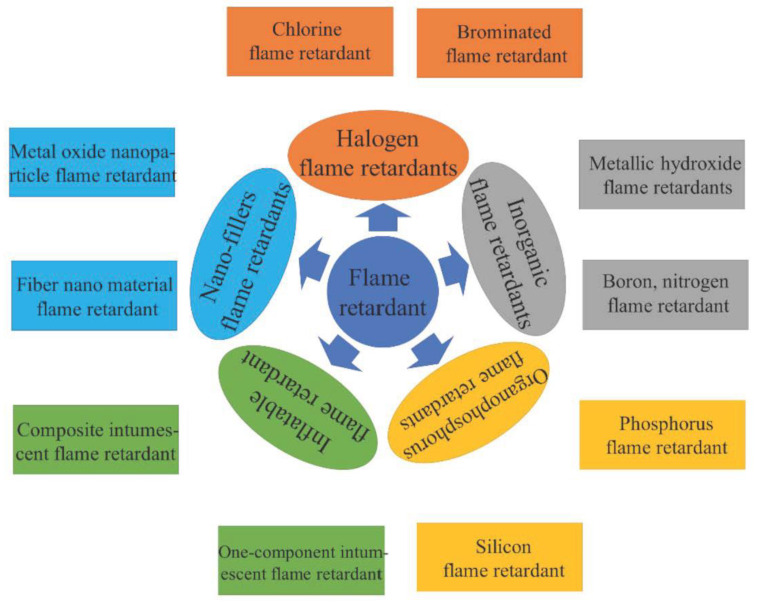
Classification of FRs for EP thermosetting materials [55]. Copyright 2022 MDPI.

Based on the way the EP matrix reacts with FRs, flame-retardant EPs are divided into two categories: reactive and additive. A number of subcategories of additive style epoxy resins containing flame retardancy are shown in Figure 10, including hybrid FR systems, intumescent FRs, nanoparticle FRs, as well as flame retardants based on phosphorus, silicon, nitrogen, and boron. Flame-retardant reactive epoxy resins are further divided into two categories: Flame-retardant hardeners as well as epoxy monomers.

#### 4.2.1. Inorganic Flame Retardant Fillers for Epoxy Resins

To increase CFRP’s flame resistance, a variety of inorganic FR fillers can be used. Among the most often used inorganic FR fillers in CFRP are red phosphorus, boron oxide or borates, ATH, Sb_2_O_3_, and MDH [56]. Greater dosage levels of inorganic FR fillers, however, could be required to achieve a high degree of fire performance in CFRP, which could have an impact on other composite characteristics or restrict design freedom [57]. To obtain the best results, inorganic FRs are usually combined with other FRs.

##### Red Phosphorus

Due to its capacity to decrease material flammability, its nontoxicity, and excellent thermal stability, red phosphorus has been utilized as a ubiquitous flame retardant in a number of applications [58]. Although red phosphorus cannot burn on its own, it can break down and release phosphorus radicals, or P2 molecules. The combustion process’s chain reaction can be broken by these radicals reacting with other free radicals. On the other hand, deadly phosphine gases, which are extremely combustible and potentially harmful to human health, are created when red phosphorus combines with moisture or water [59]. Red phosphorus and moisture can react slowly over time, but high humidity, high temperatures, or mechanical agitation can speed up the process. After adding 25 weight percent ATH and 4 weight percent red phosphorus, the EP achieved UL-94 V-0 [60].

##### Inorganic FR Synergist

Zinc borate, as well as Sb_2_O_3_, have a strong synergistic impact when coupled with halogen-based flame retardants, but they are not suitable as FRs on themselves (except halogen-containing polymers). Less flammable compounds are created in the gas phase when Sb_2_O_3_ combines with halogen-containing polymers to generate SbCl_3_ or SbBr_3_, which then react with free radicals as well as other reactive species created during epoxy combustion [61]. Zinc borate and Sb_2_O_3_ can encourage the development of a char coating on the EP composite when it is subjected to high temperatures during combustion. By physically separating flammable materials from sources of heat and oxygen, the char layer lowers heat transfer rates and stops flames from spreading [62].

##### Magnesium Hydroxide and Aluminum Trihydrate

Typical inorganic flame retardant additives in epoxy composites, ATH and MDH, enhance the fire resistance of the EP composite through a number of different processes. ATH and MDH undergo endothermic breakdown when heated during combustion. Heat is absorbed by ATH and MDH during their breakdown, reducing the composite’s temperature and delaying combustion. Water vapor, which is created when ATH and MDH react endothermically, dilutes the flammable gasses and creates a barrier that can assist in containing the spread of flames. On the epoxy composite, the breakdown of ATH and MDH results in a persistent, protective char layer. Additionally, ATH, as well as MDH, can lessen the amount of smoke produced during burning. During a fire, visibility can be improved, and the danger of smoke-related risks can be decreased by reducing the quantity of smoke generated by the combustion of water vapor as well as the development of a protective char layer [63]. Derivatives of boronic acid have been shown to increase the flame retardancy of EP/MDH composites. The composite’s LOI value rose from 21.8% to 32.5% upon the addition of boronic acid derivatives, suggesting an improvement in flame retardancy. Additionally, the composite’s UL-94 V-0 rating demonstrated its exceptional self-extinguishing properties. For pure EP, the HRR was 781 kW/m^2^. But when MH was added to the EP, the HRR dropped to 454 kW/m^2^. Additionally, the HRR dropped to 353 kW/m^2^ when boronic acid derivatives were added to the EP/MH system, suggesting that the inclusion of boronic acid derivatives increased flame retardancy and reduced the rate of heat release compared to the EP/MDH system alone [64]. APP and ATH work well together as flame retardants. EP self-extinguishes and its gross heat is reduced by 22.5% when 10 weight percent ATH and 5 weight percent APP are added. However, EP’s flame retardancy is reduced when ATH is the only ingredient added [65].

#### 4.2.2. Phosphorus Flame Retardants

##### Monomers Based on Phosphophenanthrene for EP

**Figure 11:**
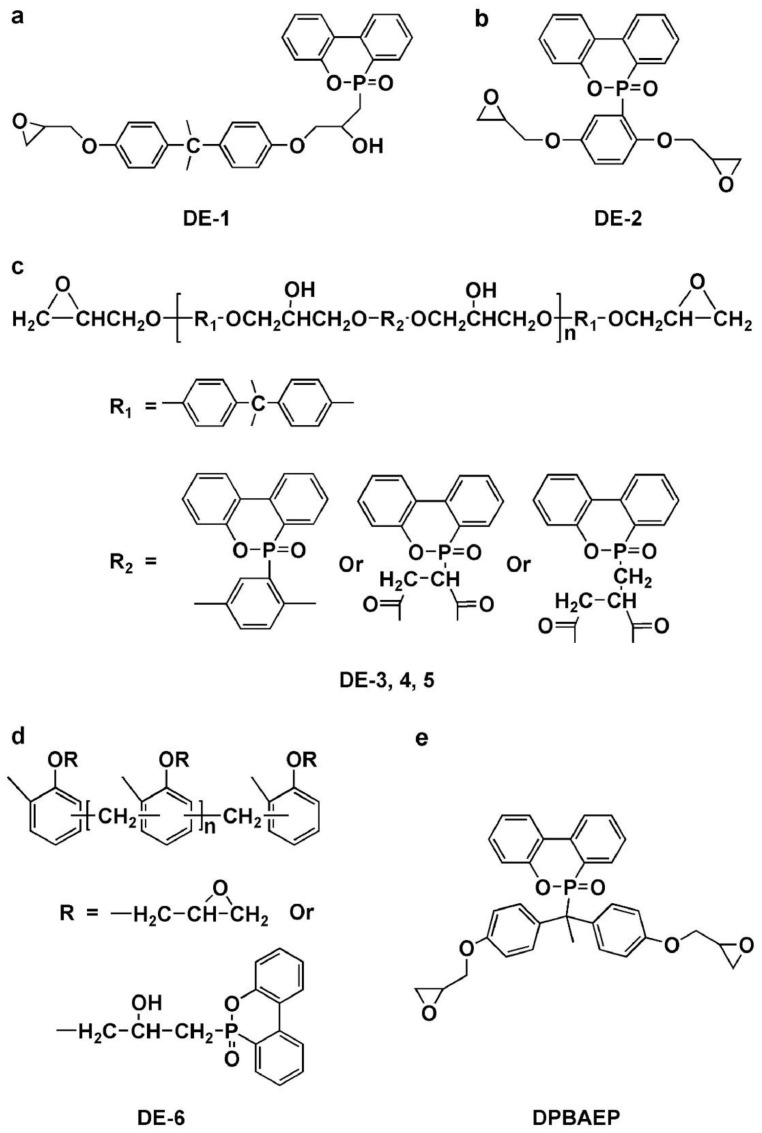
A group of phosphaphenanthrene-based EP monomers: (**a**) DE-1; (**b**) DE-2; (**c**) DE-3,4,5; (**d**) DE-6 and (**e**) DPBAEP [69]. Copyright 2021 Elsevier.

Because of its distinct phosphaphenanthrene structure, DOPO, a commercial P-containing FR, has excellent thermal stability, flame retardancy efficiency, and oxidation resistance [66]. Numerous DOPO derivatives have been created to create EP monomers containing phosphaphenanthrene because DOPO has an active P-H group. A variety of DOPO-containing EP monomers (Figure 11a–d) were synthesized by Wang et al. [67] and cured using a number of standard curing agents, including DICY, DDS, as well as PN. All EP thermosets offer exceptional flame retardancy (LOI > 27% along with V-0 rating during UL-94 testing) once the P content reaches a specific concentration. The sort of curing agents utilized has a significant impact on the final EP thermosets’ T_g_. To generate an intrinsic flame-retardant EP thermoset, Fang et al. [68] manufactured a flame-retardant EP monomer (DPBAEP) (Figure 11e) and introduced it to the TGDDM/DDS system. As the P content rises in this EP thermoset, the LOI values and UL-94 ratings rise as well, ultimately reaching 33.4% as well as V-0. To ascertain the action mechanism, the surface shape and chemical makeup of the remaining chars after the LOI test are examined using SEM along with FT-IR. An inherently flame-retardant EP thermoset is produced when the DOPO moieties interact with the EP matrix to generate a compact, including a continuous char layer. This layer can prevent heat transmission and flame propagation, including droplet formation during combustion. Because of the end-capping reaction involving P-H bonds as well as epoxy groups, it is evident that directly adding DOPO would drastically lower the T_g_ value of the EP thermoset, making it inappropriate for use in industrial settings. Conversely, DE-2 and DPBAEP are more suited for industrial applications since they have two epoxy groups that can successfully prevent end-capping reactions [69].

##### EP Monomers Based on Cyclotriphosphazene

A cyclic ring made up of alternating phosphorus and nitrogen atoms, cyclotriphosphazene exhibits exceptional thermal stability, flame retardancy, and high char production [70]. Because P and N in the ring work in concert, the cyclotriphosphazene group is commonly added to EP to enhance its FR qualities. Since a variety of substituents may replace the active chlorine atoms in commercially available HCCP by a nucleophilic substitution process, it is acknowledged as a crucial starting material for the production of cyclotriphosphazene-based EP monomers.

**Figure 12:**
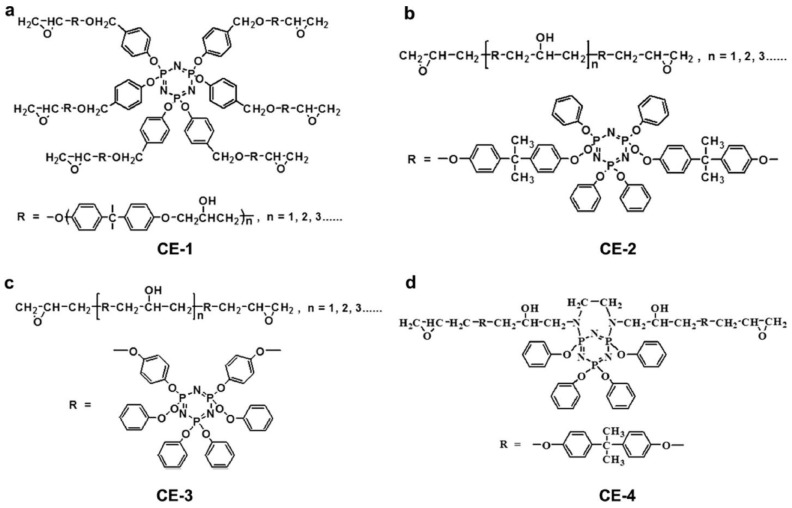
Standard cyclotriphosphazene-based EP monomers: (**a**) CE-1; (**b**) CE-2; (**c**) CE-3; and (**d**) CE-4 [69]. Copyright 2021 Elsevier.

The HGCP, a cyclotriphosphazene-containing EP monomer, was created by El Gouri et al. [71] by reacting HCCP with 2,3-epoxy-1-propanol. It was then mixed with an industrial epoxy resin (DGEBA) as well as a curing agent (DDM) to create an inherent flame-retardant EP thermoset. Because HGCP may function in the two forms of condensed and gaseous phases, the results demonstrate that 20 weight percent HGCP not only enables the final EP thermoset to attain a UL-94 V-0 rating but also greatly reduces smoke formation. In particular, (i) it encourages the development of an intumescent, P-rich char in the condensed phase, which acts as a barrier to prevent the passage of gaseous products and the transmission of heat and oxygen. HGCP breakdown produces non-combustible gases such as CO_2_, NH_3_, and N_2_, which might impede combustion by diluting combustible gases in the gaseous phase. In the meanwhile, four distinct kinds of cyclotriphosphazene-based EP monomers were made by Wang et al. [72] (Figure 12), and they were subsequently cured using a range of commercially accessible curing agents. With a UL-94 V-0 grade and comparatively high LOI values (>28%), all of the documented EP thermosets exhibit exceptional flame retardancy. Furthermore, EP thermosets with significant charring capacity (CYs > 20%) and great thermal resistance (T_gs_ > 130 °C) are produced by polyfunctional EP monomers with thermostable cyclotriphosphazene groups.

##### EP Monomers Based on Phosphate or Phosphonate

**Figure 13:**
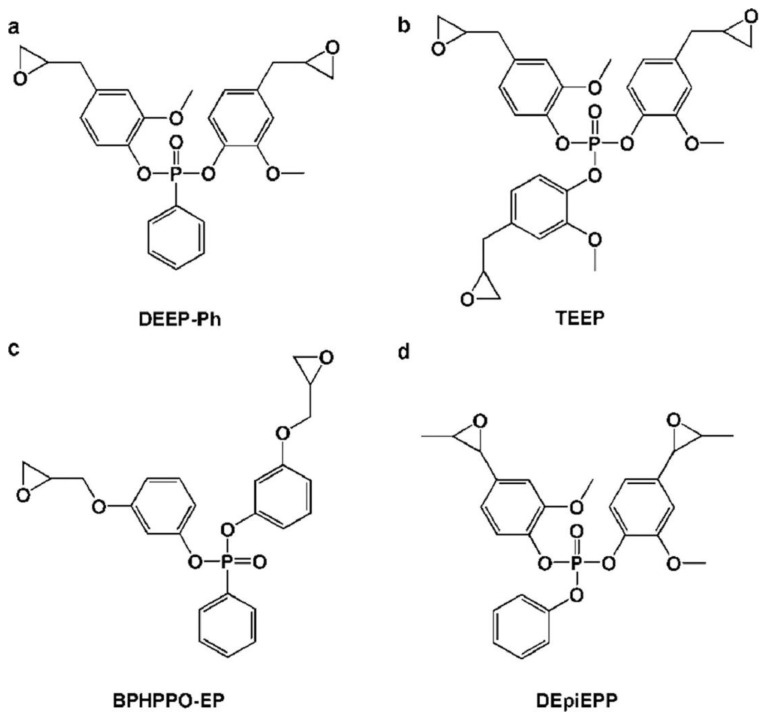
Common phosphonate-based EP monomers: (**a**) DEEP-Ph and (**c**) BPHPPO-EP); and two representative phosphate-based EP monomers: (**b**) TEEP and (**d**) DEpiEPP [69]. Copyright 2021 Elsevier.

**Figure 14:**
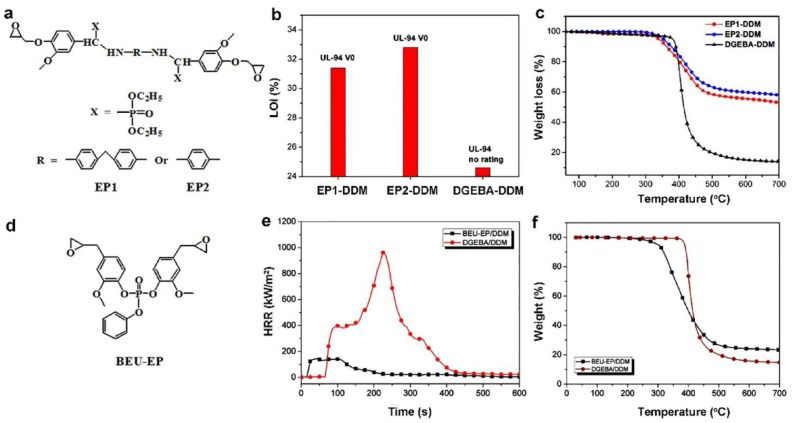
Phosphonate-based EP monomers and one phosphate-based EP monomer, along with the properties of the cured Eps: (**a**) Chemical formulas of EP1 and EP2; (**b**) UL-94 ratings and LOI values for EP1-DDM, EP2-DDM, and DGEBA-DDM samples; (**c**) TGA curves of EP1-DDM, EP2-DDM, and DGEBA-DDM samples under N_2_ flow; (**d**) chemical formula of BEU-EP; (**e**) HRR curves of BEU-EP/DDM and DGEBA/DDM samples; and (**f**) TGA curves of BEU-EP/DDM and DGEBA/DDM samples under N_2_ flow [69]. Copyright 2021 Elsevier.

Moreover, EP thermosets with inherent flame resistance have been produced using phosphonate- as well as phosphate-based EP monomers (Figure 13) [73]. For instance, Figure 14a displays the chemical structures of two kinds of biomass vanillin-derived phosphonate-based EP monomers (EP1 as well as EP2) that were recently produced by Ma et al. [74]. Two bio-based flame-retardant EP thermosets (EP1-DDM and EP2-DDM) are produced by curing with DDM. The EP1-DDM and EP2-DDM systems receive a V-0 rating with LOI values of 31.4% as well as 32.8%, respectively, but the DGEBA-DDM technology fails the UL-94 test despite a low LOI value of just 24.6%, as seen in Figure 14b. Additionally, compared to DGEBA-DDM, EP1-DDM as well as EP2-DDM both have substantially higher CYs (Figure 14c) as well as generate a more continuous overall intumescent char, which accounts for the noticeably better flame-retardant performance. Because of its stiff structure, vanillin-derived phosphonate-based EP has a high T_g_ of about 214 °C, a tensile strength of about 80.3 MPa, as well as a tensile modulus of around 2709 MPa. However, because of the thermally unstable diethyl phosphite, and this has also been seen in other diethyl-phosphite-containing EPs systems, they have poor thermal stability, as evidenced by a low T5% value (<300 °C) [75].

One bio-based EP thermoset was created by Liu et al. [76] using a phosphate-based EP monomer produced from eugenol (BEU-EP) (Figure 14d). When compared with the control DGEBA/DDM sample at a heat flux of 50 kW/m^2^, the produced BEU-EP/DDM shows remarkable flame retardancy, featuring an LOI of 38.4%, a UL-94 V-0 rating, along with an ∼85.1% reduction in PHRR (Figure 14e). Likewise, the BEU-EP/DDM sample exhibits higher char production (Figure 14f), which is principally in charge of enhanced flame retardancy.

#### 4.2.3. Silicon Flame Retardants

##### Siloxane

**Figure 15:**
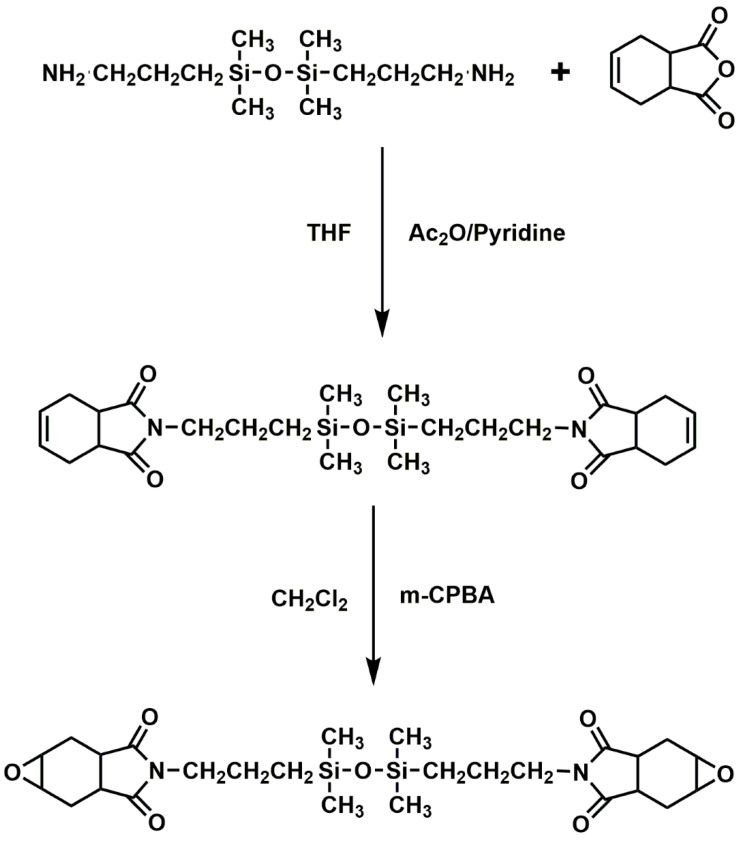
Synthesis of the BISE [79]. Copyright 2007 Elsevier.

Alkyl, alkyl substituent, as well as phenyl, are side chains of siloxanes, which are significant silicon FRs with the Si-O-Si link as the primary chain. As non-toxic, low-smoke, eco-friendly, and highly effective FRs, siloxanes have drawn a lot of interest in recent years [77]. Low surface area siloxanes may move from the polymer to the surface during combustion and combine with the polymer to create a thick and stable layer of O-insulating carbon. This stops the products of combustion from escaping as well as stops the composites from thermally decomposing [78]. BISE, a novel cycloaliphatic epoxy composite with siloxane, was created (Figure 15) [79]. BISE demonstrated better mechanical and thermal qualities than traditional cycloaliphatic EPs. Moreover, BISE’s remarkable dielectric, along with moisture resistance, was caused by the hydrophobic and low polarity siloxane sections in the epoxy backbone. Scientists and engineers are interested in the imide group because of its exceptional mechanical strength, thermal stability, as well as interaction using EP at high temperatures. An innovative EP modified with siloxane and imide that cures with silica-containing dianhydride was developed [80]. The durability, mechanical qualities, and thermal stability of the EP were greatly enhanced by the siloxane along with imide groups. It was noteworthy that these changed systems’ T_g_ values were higher than 160 °C. The siloxane group’s conversion to flake silica as a char residue during pyrolysis was the cause of the high thermal stability, which successfully prevented heat transmission in the polymer.

**Figure 16:**
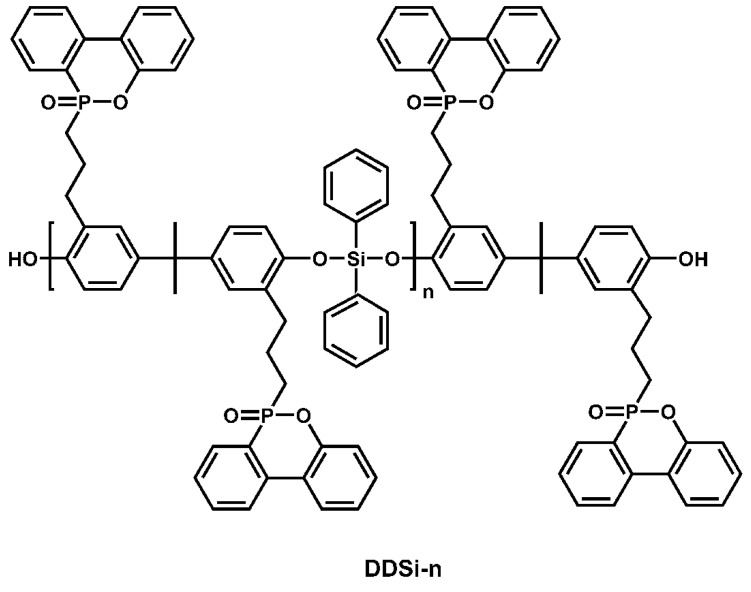
Chemical structure of contrasting DDSi-n [81]. Copyright 2018 ACS.

Silicanes are commonly used with other very powerful flame-retardant chemicals to increase their flame-retardant efficacy. Bifunctional group-containing new macromolecules (DDSi-n) were produced (Figure 16) and evaluated for combustibility on EPs [81]. The results showed that the synergistic FR impact of the phosphinophenanthrene, including phenylsiloxane groups, enhanced the mechanical characteristics and flame retardancy of EPs when DDSi-n macromolecules were added. The classification was UL-94 V-1, the LOI value was 35.9%, as well as the DDSi-1 content was 8 weight percent. While the UL-94 level rose as the degree of DDSi-n polymerization grew at the same rate of addition, the epoxy composite’s LOI value fell. As DDSi-1 content rose, both pk-HRR along with THR sharply declined. As the amount of DDSi-n or the degree of polymerization decreased, the effect on the strength of DDSi-n/EP rose. Later research used DDSi-1 to create two cluster-like molecules (TriDSi along with TetraDSi), which were then added to EPs [82]. The mechanical qualities and flame resistance of EPs were enhanced with the incorporation of TriDSi along with TetraDSi. The epoxy composites completed the UL-94 V-0 test with a weight percentage of 6; the LOI values were 35.2% for TriDSi and 36.0% for TetraDSi; the impact strength rose by 133% for TriDSi and 123% for TetraDSi, respectively; along with the pk-HRR and THR decreased in comparison to neat EP. The thorough examination of the experimental data showed that EP’s mechanical with flame-retardant qualities were derived from the segmer-aggregation impact of cluster-like compounds and the synergistic effect of phosphophenanthrene as well as siloxane groups.

##### Silica

One frequent filler used to alter EP is silica. Incorporating silica into polymers may encourage the development of carbon layers and enhance resistance to oxidation, which not only offers flame retardancy but also enhances mechanical qualities, heat resistance, and processability [83]. One crucial strategy for creating eco-friendly flame retardants is the creation of innovative silicon-based compounds.

Because of its low thermal expansion coefficient, high loading, and fluidity, spherical silica has attracted a lot of attention. After creating spherical silica particles with a homogenizer, S.-E. Hou et al. [84] introduced them to EP as an additive. Mechanical characteristics, coefficient of thermal expansion, and thermal stability were examined in relation to different percentages of spherical silica. The mechanical characteristics and early decomposition temperature of the composites were greatly enhanced by the addition of silica. Additionally, the composites showed the best mechanical and thermal stability at 30 weight percent spherical silica concentration.

**Figure 17:**
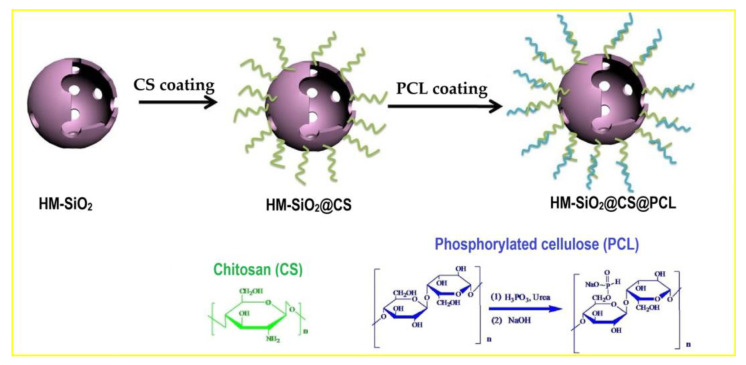
Synthesis of HM-SiO_2_@CS@PCL [85]. Copyright 2018 Elsevier.

Hollow mesoporous silica (HM-SiO_2_) (Figure 17) was coated with chitosan (CS)/phosphorylated cellulose (PCL) to create a green multi-element silica derivative (HM-SiO_2_@CS@PCL), which was then added to the EP matrix as an FR. The findings demonstrated the strong flame retardant effectiveness of HM-SiO_2_@CS@PCL. Char residue rose from 5.0% to 17.8% at 700 °C. TSP and pk-HRR declined by 18.7% and 51%, respectively. The creation of a thick layer of carbon that acted as a barrier enhanced the flame retardancy of EP/HM-SiO_2_@CS@PCL.

##### POSS

POSS are nanocompounds that range in size from 1 to 3 nm in three dimensions, with 1.5 nm separating R groups as well as 0.5 nm separating Si atoms. SinO_3n/2_R_n_, or T_n_R_n_, is the generic formula [56]. The most prevalent version, Si_8_O_12_R_8_ or T_8_R_8_, is a cage frame composed of twelve oxygen atoms along with eight silicon corner atoms. Every silicon corner atom may be linked to a group (R), and depending on the necessary material qualities, the characteristics and quantity of R groups can be deliberately adjusted [86]. Interest in utilizing POSS has increased due to their adaptability [87]. Numerous benefits of silica along with siloxane, including mechanical qualities, low toxicity, dissolution, ease of modification, flame retardancy, and thermal as well as chemical stability, are combined in the structure of POSS [56]. POSS-based polymers may develop a carbon coating on their surfaces at high temperatures, which would stop heat and oxygen from transferring [88]. There have been reports of many EP/POSS systems as of right now [89].

**Figure 18:**
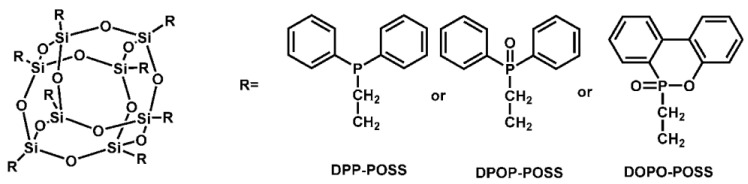
Structures for DPOP-POSS, DOPO-POSS, and DPP-POSS [90]. Copyright 2016 Elsevier.

Octa-vinyl POSS was combined with DPP, DPOP, and DOPO to create a sequence of phosphorus-containing POSS (Figure 18) [90]. Flame-resistant EP composites with a 5-wt.% composition were made using DOPO-POSS, DPOP-POSS, along with DPP-POSS. The results showed that EPs’ flame retardancy may be increased by all three flame retardants. EP/DPP-POSS passed the UL-94 V-0 test, whereas EP/DPOP-POSS with EP/DOPO-POSS passed the UL-94 V-1 test. The LOI values were 33.2% (EP/DPP-POSS), 29.3% (EP/DPOP-POSS), as well as 30.0% (EP/DOPO-POSS). While the residues rose from 3.5% (neat EP) to 20.2% (EP/DPP-POSS), 17.9% (EP/DPP-POSS), along with 19.1% (EP/DPP-POSS), respectively, the pk-HRR along with THR sharply declined. It was suggested that these flame retardants would function in both gaseous and condensed forms. By intensifying charring in the condensed phase and releasing phosphorus volatiles in the gas phase, the flame retardant effect was accomplished.

**Figure 19:**
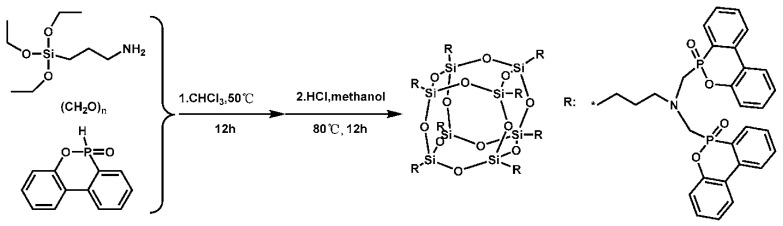
ODMAS’ synthetic route [56]. Copyright 2020 MDPI.

After being synthesized (Figure 19) as well as combined with EPs, a new halogen-free FR (ODMAS), including DOPO along with POSS, was evaluated for flame retardancy [91]. Experiments have demonstrated the fire resistance of EP/ODMAS composites. The LOI value of 35.5%, as well as UL-94 V-0 rating, were attained even with a low ODMAS concentration of 5 weight percent. As the amount of ODMAS in EP/DOMAS composites grew, so did the production of char. Furthermore, because ODMAS is highly soluble in EP, its inclusion in the matrix enhanced mechanical characteristics.

#### 4.2.4. Nitrogen Flame Retardant

Nowadays, the most common nitrogen-containing flame retardants found in epoxy resins include urea, guanidine, melamine, and ammonia, among various hardener derivatives. According to the flame retardant process, undamaged melamine molecules split apart in the gas phase to produce cyanamide, which can then undergo pyrolysis to produce poor fuels that are rich in nitrogen [92]. When employed as EP flame retardants alone, melamine and its phosphorus-containing anionic salts (APP and MPP) have weak flame retardant qualities. It is best to use melamine in combination with other flame retardants. An example of a system that uses APP is an intumescent flame retardant [93]. Additionally, it has been extensively documented that combining nitrogen-containing flame retardants with flame retardants that include other elements, including phosphorus [94], sulfur [95], as well as silicon [96], in epoxy resins produces EP composites with exceptional flame retardancy and other qualities. In the synergistic flame retardant combination of phosphorous and nitrogen, HCCP, as well as its derivatives, are common FRs [97]. By altering the structure of cyclotriphosphonitrile, additional FRs, such as triisopropyl borate [98], DOPO [99], along with bisphenol-S [100], may be added, enabling the use of FRs with superior flame retardancy in EP composites.

#### 4.2.5. Intumescent Flame Retardants

**Figure 20:**
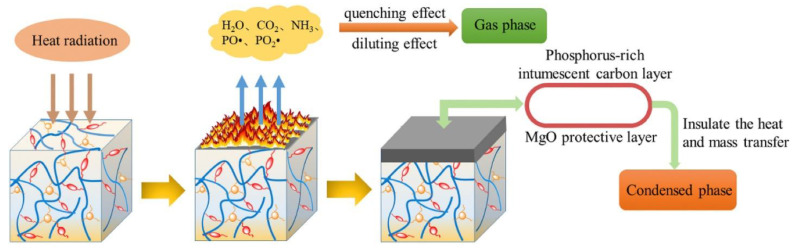
EP thermosets introduce new intumescent flame retardant mechanisms [107]. Copyright 2021 Elsevier.

Because of their low toxicity, excellent thermal stability, and halogen-free characteristics, intumescent flame retardant substances have recently been demonstrated to emit less smoke when burnt [101]. This has generated a lot of interest in research. IFR is unique in that it can facilitate the development of an expandable foam-like char layer in the early phases of thermal breakdown and polymer combustion, serving as a useful physical barrier to lessen the blockage of gas and heat exchange [102]. APP is a conventional FR that may provide IFR/EP systems using gas and acid [103]. Long chains of APP start to decompose at temperatures between 240 and 380 °C, releasing phosphoric acid, molecular nitrogen, along with ammonia. The expansive flame retardant’s name comes from the char layer that forms when phosphoric acid is released, which also encourages the “expansion” of the polymer substance and prevents it from burning any further. Additionally, when the emission of non-combustible gases declines, so does the concentration of oxygen with combustible gases. Therefore, adding a small amount of APP to epoxy resin can result in superior flame-retardant qualities [104]. However, APP has a number of disadvantages, such as low load and incompatibility with the epoxy matrix. Microencapsulation technology has drawn a lot of interest as a solution to the problems with acid precursors like APP [105]. Additionally, the use and creation of novel IFRs on epoxy resins have been the focus of study in recent years [106]. An example of a flame retardant system is shown in Figure 20.

#### 4.2.6. Hybrid Flame Retardant Systems

Despite their numerous advantageous qualities, nanomaterials such as CNTs, graphene, LDH, and MXene are challenging to completely and uniformly disperse into epoxy resin utilizing a straightforward mixing technique. Moreover, a single nanofiller’s low flame retardant efficacy led to the creation of a hybrid flame retardant system. Organic–inorganic hybrids, known as a hybrid flame retardant system, mix inorganic nanofillers with organic flame retardants (such as phosphorus) through intermolecular interactions, ionic bonds, covalent bonds, and hydrogen bonds. They are utilized in EPs to produce high-performance, flame-retardant EP composite products.

**Figure 21:**
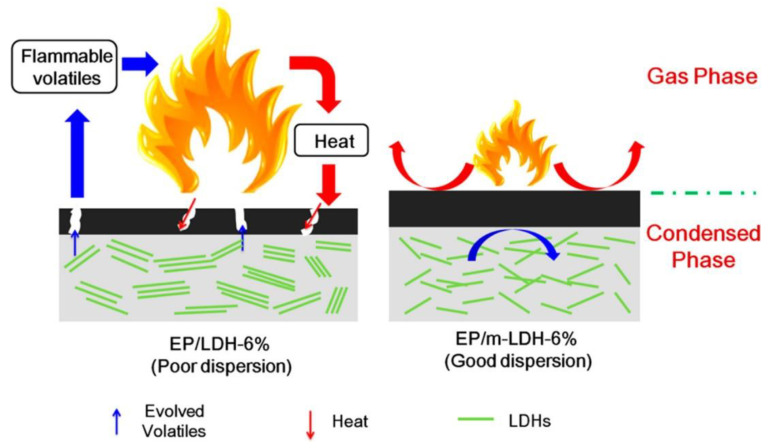
A schematic illustration of EP/m-LDH’s flame retardant mechanism [110]. Copyright 2015 ACS.

Because of its poor dispersion and limited FR efficacy in EP matrices, MMT, a well-known nanoflame retardant, can also be utilized in hybrid flame retardant systems. Zhang et al. [108] recently altered MMT with DOPO to increase its flame-retardant effectiveness and dispersion in an EP matrix. Because of the synergy between DOPO as well as MMT, the EP composite (EP/MMT-DOPO) has outstanding FR qualities and effectively compensates for each material’s weaknesses. Sulfonates or fatty acid salts are frequently employed as intercalators because of the strong contact and polarity of LDH layers, which impact both the mechanical qualities of the epoxy matrix and the dispersion of the layers. They might be surfactants or sulfonates that serve as LDH intercalators. Sulfonate, a bio-based surfactant, was created by Wang et al. [109] along with proved to be an efficient dispersion and LDH modifier. As seen in Figure 21, evenly distributed m-LDH creates a dense, continuous residue that acts as a mass transfer barrier to keep flammable gasses from entering the interior and as a superior insulator to shield the underlying substrate from external heat radiation.

Additionally, the design, along with the synthesis of phosphorous intercalators, is a research priority of LDH flame retardant systems since phosphorus may enhance the generation of char during burning, enhancing the FR properties of EP composites [109].

#### 4.2.7. Bio-Based FRs

In order to construct bio-based formulations, it is essential to synthesize FR bio-epoxy resins from renewable resources with sufficient mechanical qualities equivalent to DGEBA.

In terms of TTI, pHRR, along with THR, Miao et al. [111] evaluated the flammability of bio-based EUFU-EP in comparison to commercial DGEBA treated with methyl MHHPA. In addition to having the same TTI values as the systems under study, EUFU-EP performed better in terms of flame retardancy since its pHRR and THR values were lower. The main cause of this was the EUFU-EP resin’s packed aromatic structure, which raised the char content because of its higher T_g_. Furan, along with aromatic structures in the EUFU-EP backbone, improved the mechanical characteristics of EUFU-EP/MHHPA even though its crosslink density was shorter than that of the EUFU-EP/MHHPA system.

Similarly, biologically produced DGED cured with DDM was shown to be more reactive than petroleum-based DGEBA by Dai et al. [112]. More unsaturated double bonds in the DGED structure throughout the curing reaction were thought to be the cause of the higher reactivity. Consequently, DGED’s viscosity rose more quickly than DGEBA’s, and the DGED/DDM system’s thermomechanical characteristics performed better than those of the DGEBA/DDM system. During combustion, DGED created char at the surface, protecting the lower layers from heat transmission and causing a little flame to self-extinguish in three seconds. It is challenging to comprehend the flame behavior of the suggested system while taking into consideration the resistance of elements from the condensed phase that influenced flame retardancy since complementing flame experiments, such as cone calorimetry, were not conducted.

By joining two eugenol molecules with epoxidizing terminal groups (DEU-EP), Wan et al. [113] created bio-epoxy resins, producing a product with a high bio-based content of almost 70%. DDM was used to cure this monomer. Using a model-free isoconversional method, the authors examined the cure kinetics mechanism of bio-epoxy and found that the inclusion and configuration of eugenol building blocks in the chain backbone significantly affected the cure behavior of DEU-EP/DDM systems. This led to improvements in mechanical characteristics and flame resistance, including the ability to char at high temperatures. DEU-EP/DDM resin left 38% char at 800 °C, which is almost twice as much as the DGEBA/DDM system. In addition, DEU-EP’s pHRR (201 kW/m^2^) and THR (16.3 kJ/m^2^) were much lower than DGEBA’s. DEU-EP demonstrated intrinsic flame retardancy by achieving self-extinguishment in 10 s. The storage modulus of DEU-EP dropped sharply at higher temperatures, reaching a T_g_ of 114 °C. The polymer chains’ enhanced free volume, along with molecular mobility, were the cause of this. Later, the same group [114] created TPEU-EP, which has a complete aromatic ester backbone and increased flame retardancy. Because of its aromatic nature, TPEU-EP showed better mechanical qualities than commercial DGEBA, even though it had a lower crosslinking density. Concrete proof of TPEU-EP’s possible use as a DGEBA substitute was supplied by cone calorimetry as well as burning analysis. For mass manufacture, the TPEU-EP synthesis method was economical. A survey of the literature indicates that while the synthesis of FR bio-epoxy is still in its infancy, chemically implanted FR components in bio-epoxy were substitutes for naturally occurring FR bio-epoxy.

#### 4.2.8. Phosphorous-Containing FR

A number of studies have examined the flame-retardant properties of compounds comprising phosphorus, which are mainly enhanced by the production of char in the condensed phase [115]. Diglycidyl mono-phosphonated phloroglucinol reactive FR, which originates from renewable phloroglucinol materials, was incorporated in P3EP by Menard et al. [116]. They found that the phosphorous FR degraded the thermal resistance of epoxy resin; the phenomenon was explained by the plasticizing function of the FR. Yet, PCFC findings and char content tests showed that the flammability of phosphorous flame retardants incorporated into bio-epoxy was greatly increased. It should be mentioned, nonetheless, that in order to completely comprehend the behavior of the flames in this system, additional flammability experiments were mostly necessary. IA and DOPO were combined to create a phosphorus-containing bio-epoxy resin (EADI) in a different study [117]. This bio-based FR was then employed in the DGEBA system. It was found that the mechanical and thermal characteristics of DGEBA/EADI were comparable to those of DGEBA systems and that the bio-based systems’ flame retardancy was significantly improved in terms of LOI, char content, and burning time.

#### 4.2.9. Silicon-Containing FR

By creating residues in the condensed phase along with releasing radicals into the vapor phase, silicon compounds increase the flame retardancy of EPs [118]. EPEU was connected to silicon-containing bridges of different lengths along with chemical compositions by Li et al. [119]. It was discovered that the manufactured silicon-containing bio-epoxy resin was noticeably more combustible than the brand-name DGEBA. The recorded LOI for DGEBA was 22.8%, while for bio-epoxy resins containing phenyl siloxane, it rose to 31%. This was made clear by the movement of Si-O to the surface along with the carbonation of phenyl groups, which produced an ablative layer that stopped fuel and oxygen from entering the combustion layer. Additionally, as the length of the siloxane linker rose, the generated silicon-containing bio-epoxy’s viscosity reduced, aiding in the EP curing reaction.

## 5. Interfacial Bonding Between CFRPs and Steel in Hydrogen Storage Materials

**Figure 22:**
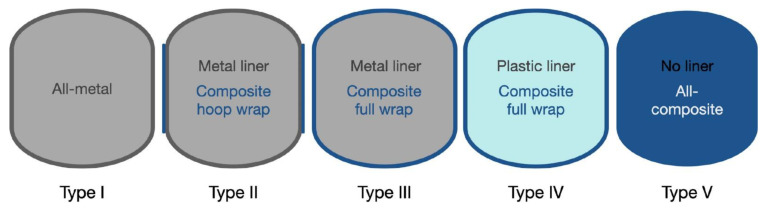
Five types of hydrogen storage tanks [5]. Copyright 2024 Elsevier.

Compressed hydrogen is increasingly being recognized as a viable alternative to traditional energy sources [1]. In recent years, it has garnered significant interest as a clean fuel option. Consequently, various hydrogen storage methods have been developed to meet this growing demand. Storage tanks play a crucial role in advancing hydrogen storage and transportation technologies [2]. Hydrogen vessels are categorized into four primary types: type I, type II, type III, IV, and type V (Figure 22) [120].

**Figure 23:**
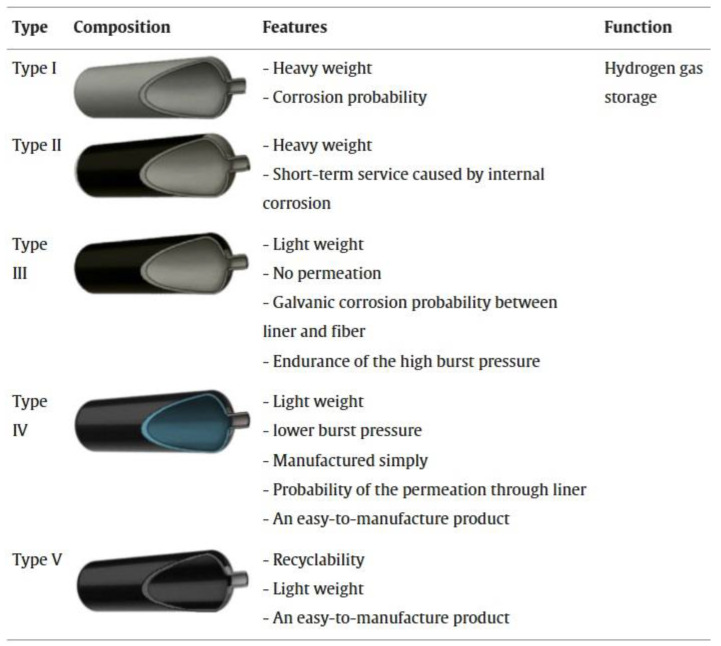
Types and generations of hydrogen storage tanks, as well as their related features [120]. Copyright 2024 Elsevier.

**Figure 24:**
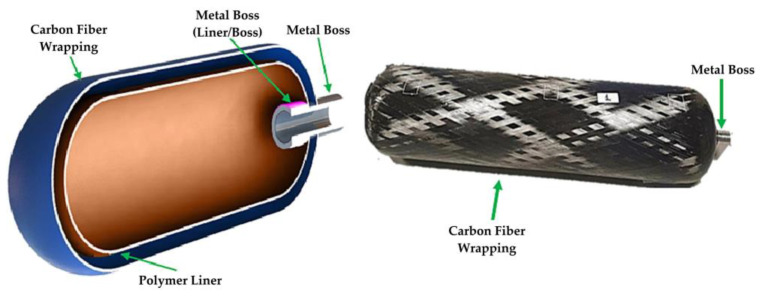
A pressure vessel of type IV for hydrogen [120]. Copyright 2024 Elsevier.

Figure 23 outlines the distinct characteristics of each type of hydrogen vessel. Type III hydrogen tanks demonstrate certain limitations when compared to type IV, particularly in vehicle applications, where factors such as cost, weight, and efficiency are concerned [2]. In contrast, type IV hydrogen tanks feature a plastic barrier layer that offers several advantages over type III tanks, including lower cost, resistance to corrosion, enhanced fatigue resistance, durability, and reduced weight [2]. Specifically, type IV tanks consist of a polymer liner, an outer layer made of carbon fiber composite, and a metallic nozzle (Figure 24). The polymer liner effectively prevents hydrogen permeation, while the carbon fiber composite layer enhances the mechanical strength of the tank [121]. Additionally, hydrogen gas is introduced into the tank via the metallic nozzle.

Given that hydrogen is highly permeable and that type IV high-pressure storage vessels operate under extremely high pressures, the interface between the polymer liner and the metallic nozzle is a critical area. This location is particularly susceptible to hydrogen diffusion. Consequently, the interface remains one of the significant vulnerabilities of type IV high-pressure vessels, despite numerous studies conducted on the subject. A variety of investigations are underway to improve metal-polymer adhesion properties through the application of different techniques [122].

Over the years, several theories of adhesion have been proposed, including the chemical bonding principle, the diffusion principle, the electrostatic (contact charging) theory, the wetting (adsorption) theory, and the mechanical interlocking theory. These theories suggest that various parameters affecting adhesion between two materials can influence the properties of their interface, thereby enhancing adhesion overall [120].

M. Ahmadifar and colleagues [120] investigated how surface treatments affect fatigue characteristics, which are critical for improving the reliability and safety of hydrogen storage systems. With advancements in manufacturing techniques such as rotational molding, this study emphasizes the possibility of improved bonding methods that eliminate the need for welding, making these tanks lighter and more efficient.

The key findings concerning the fatigue characteristics of metallic boss-polymer liner adhesion reveal that adhesion strength significantly improves with specific surface treatments, particularly sandblasting, resulting in an apparent shear strength increase of approximately 30% when compared to untreated surfaces. The study found that the metal-polymer interface has a linear relationship with load amplitude during fatigue testing, with fatigue strength consistent up to 10 MPa, indicating resilience against damage and fracture under cyclic loading. The rotational molding process improves the bonding between metallic components and polymer liners by allowing for direct integration of the metallic nozzle into the polymer matrix at high temperatures, resulting in better interfacial adhesion via mechanical interlocking and improved surface roughness. This process not only improves the microstructure of the interface but also reduces the risk of hydrogen permeation, resulting in a more robust and reliable hydrogen storage solution, which is critical for automotive applications.

**Figure 25:**
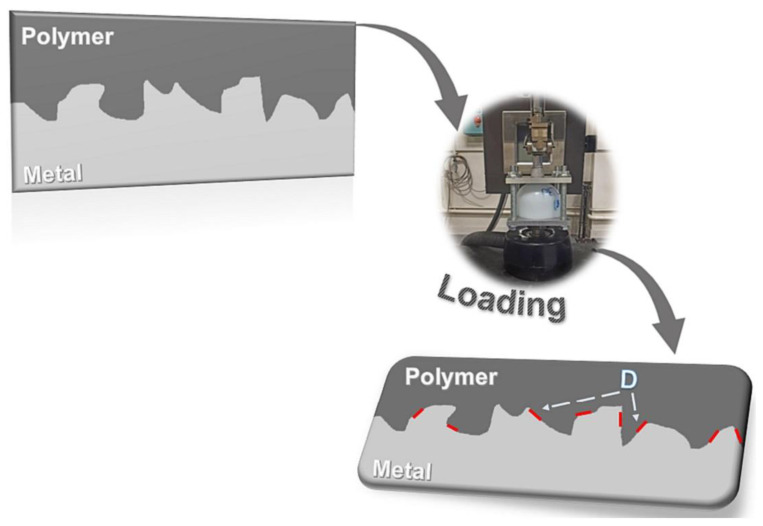
Possible joining mechanisms for metal–polymer hybrids [120]. Copyright 2024 Elsevier.

Possible joining mechanisms in metal–polymer hybrids include a combination of physical and chemical interactions that improve the adhesion between the two materials (Figure 25). Mechanical interlocking is a key mechanism in which surface treatments such as sandblasting create a roughened texture on the metal surface, allowing the polymer to grip and bond better during the molding process. Chemical bonding can also occur at the interface by forming covalent or hydrogen bonds, which can significantly strengthen adhesion. Diffusion mechanisms also play a role, as polymer chains can penetrate the metal surface, resulting in more intimate contact and stronger bonds. Furthermore, surface treatments can improve the wettability of metal surfaces, allowing for better polymer flow and coverage during manufacturing.

## 6. Challenges

### 6.1. Challenges of Gas Permeation in Epoxy Resin Materials for Hydrogen Storage Applications

Gas permeation in epoxy resin materials for hydrogen storage applications presents significant challenges that impact the effectiveness and safety of storage systems. While epoxy resins offer excellent mechanical properties and chemical resistance, they typically exhibit high gas permeability, allowing hydrogen to diffuse more easily than desired. This permeability can lead to hydrogen leakage, posing safety risks and reducing storage efficiency. The presence of microvoids and defects within the resin further facilitates gas permeation, compromising the integrity of storage vessels. Moreover, hydrogen interaction with the epoxy matrix can result in chemical degradation and embrittlement over time. The intrinsic free volume of epoxy resins contributes to increased hydrogen diffusion rates [5]. Incorporating gas-barrier fillers, such as graphene nanoplatelets [123] and montmorillonite (MMT) [124], is crucial for enhancing hydrogen barrier properties. However, achieving optimal dispersion and interfacial bonding between fillers and resin is critical, as poor compatibility can reduce effectiveness and increase permeability. Temperature-induced strain during operation can also affect the integrity of the resin and barrier films, potentially creating interfacial defects that compromise overall performance. Addressing these challenges requires innovative strategies in material design and processing. Enhancing the gas barrier properties of epoxy resins is essential for developing reliable and efficient hydrogen storage solutions [125]. This may involve optimizing resin formulations, improving filler dispersion techniques, and developing novel composite structures that minimize gas permeation while maintaining the desirable mechanical properties of epoxy-based materials [126].

### 6.2. Challenges Associated with CFRP Composite Porosity and Its Influence on Fire Retardancy

Porosity in CFRP composites poses significant challenges, including compromised mechanical properties, reduced durability, and diminished fire retardancy. Voids can create stress concentrations, increasing the likelihood of failure under load, while trapped air within these voids can enhance combustion rates and smoke production during a fire, leading to more intense fires. Additionally, uneven porosity distribution can result in unpredictable thermal degradation, further jeopardizing structural integrity. Effective management of porosity through careful processing and curing techniques is essential for improving fire resistance and ensuring reliability in critical applications. In the context of autoclave lamination, a common method in aerospace manufacturing, porosity often arises from trapped air and absorbed moisture, leading to defects that can reduce mechanical properties and result in part rejection. To address this, Dei Sommi et al. [127] developed a multiphysic model aimed at identifying and mitigating water-generated porosity by modifying process parameters rather than focusing on void growth. This model, validated through its application to epoxy matrix CFRP laminates, predicts conditions leading to porosity during autoclave curing and provides strategies for prevention. It simulates the distribution of water concentration, temperature, resin pressure, and degree of reaction across laminates, guiding the optimization of process parameters to enhance composite quality while minimizing defects. Key modifiable parameters include bleeder thickness, curing temperature, pressure profiles, and initial moisture content in the resin. The research findings confirm that the model effectively predicts conditions leading to porosity, demonstrating that factors like residual moisture and water vapor pressure significantly influence porosity levels. Validation through micro-CT analysis showed that higher humidity correlates with increased porosity, underscoring the importance of optimizing process parameters to improve the quality and performance of CFRP composites.

### 6.3. Effect of Manufacturing Technologies on the Flame Retardancy of CFRP Composites

The impact of manufacturing technologies on the flame retardancy of CFRP composites is substantial, as methods like vacuum infusion and hand lamination significantly affect the distribution and effectiveness of flame retardants such as ammonium polyphosphate (APP). Toldy et al. [128] observed that vacuum infusion can cause a pronounced accumulation of APP near the infusion site, leading to uneven distribution that compromises fire performance in deeper layers of the composite. In contrast, hand lamination typically results in a more uniform distribution of additives, thereby enhancing overall flame retardancy. Furthermore, the choice of manufacturing technology influences the fiber content and viscosity of the resin, which in turn affects the thermal properties and ignition resistance of the composites. Optimizing the manufacturing process is, therefore, essential for improving the fire performance of CFRP composites. The study compares two primary manufacturing techniques: vacuum infusion, which allows efficient resin impregnation but may result in uneven flame retardant distribution due to filtration effects, and hand lamination, followed by hot pressing, which offers a more controlled layering environment and better additive distribution. The addition of APP significantly enhances the flame retardancy of CFRP composites by reducing pHRR and TTI, as it forms a protective char layer during combustion that slows the burning process. However, the effectiveness of APP is contingent upon its distribution within the composite; uneven distribution can compromise flame retardancy. Cone calorimeter tests revealed that vacuum-infused composites exhibited lower pHRR and longer ignition times compared to those made by hand lamination, indicating improved flame retardancy due to more effective APP accumulation. These findings highlight the critical role of manufacturing technology in optimizing the fire resistance of CFRP composites.

## 7. Conclusions and Future Perspective

Recent advances in the fire safety of CFRP composites for hydrogen storage tanks have shown significant progress in the thermal resistance and mechanical stability of the materials under extreme conditions. These composites are widely used due to their lightweight and robustness, but their flammability remains a major issue, especially under high heat. Challenges include the thermal degradation of composites at high temperatures, which can weaken the structure and lead to explosion risks, as well as the integration of flame retardants that do not alter the mechanical properties of the materials. Furthermore, constraints related to the compatibility of different additives and the protection of the tanks against severe temperature and pressure conditions exacerbate the design challenges. Future perspectives include the exploration of nanomaterials, such as graphene nanoplatelets, which could provide enhanced fire protection while strengthening the structure of the composites. In addition, more advanced thermal and mechanical models are needed to accurately predict tank behavior in fire and accident situations. Finally, special attention must be paid to the long-term durability of tanks, facing temperature and pressure cycles, to ensure the safety and reliability of long-term hydrogen storage.

## Abbreviations

EPs	Epoxy resins
EP	Epoxy resin
FTIR	Fourier transform infrared microscopy
PHRR	Peak heat release rate
UL 94	Underwriters Labs 94 test
LOI	Limiting oxygen index
DSC	Differential scanning calorimetry
THR	Total heat release
DTA	Differential thermal analysis
HRR	Heat release rate
Py-GC-MS	Pyrolysis gas chromatography/mass spectrometry
SDT	Smoke density test
MHHPA	Hexahydrophthalic anhydride
T_g_	Glass transition temperature
DGED	Diglycidyl ether of daidzein
DDM	4,4′-diaminodiphenylmethane
TPEU-EP	eugenol-based bifunctional epoxy resins
FR	Flame retardant
FRs	Flame retardants
P_3_EP	Triglycidyl phloroglucinol
PCFC	Pyrolysis combustion flow calorimetry
IA	Itaconic acid
TG	Thermogravimetric analyzer
EPEU	Epoxidized eugenol bio-epoxy
DICY	Dicyandiamide
DDS	4,4′-Diaminodiphenyl sulfone
PN	Phenol novolac
DDSi-n	Phosphinophenanthrene/phenylsiloxane bifunctional groups
POSS	Polyhedral oligomeric silsesquioxanes
DPP	Diphenylphosphine
DPOP	Diphenylphosphine oxide
TSR	Total smoke released
HCCP	Hexachlorocyclotriphosphazene
APP	Ammonium polyphosphate
IFR	Intumescent flame retardant
MS	Mass spectrometry
CFRPs	Carbon fiber-reinforced epoxy composites
FAR	Failure analysis report
CF	Carbon fiber
ATH	Aluminum trihydrate
TTI	Time to ignition
MDH	Magnesium hydroxide
Sb_2_O_3_	Antimony trioxide
RT	Room temperature
XPS	X-ray photoelectron spectroscopy
TTI	Time to ignition
